# Designing RNA sequencing experiments: A practical guide to reproducible gene expression analysis

**DOI:** 10.1016/j.csbj.2025.12.015

**Published:** 2025-12-18

**Authors:** Kamil Antoszewski, Klaudia Chmielewska, Karolina Jagiello, Tomasz Puzyn

**Affiliations:** Laboratory of Environmental Chemoinformatics, Faculty of Chemistry, University of Gdansk, Jana Bażyńskiego 8, Gdańsk 80-309, Poland

**Keywords:** RNA sequencing, Transcriptomics, Bulk RNA-seq, Gene expression, Multi-omics

## Abstract

RNA sequencing (RNA-seq) has become a cornerstone of modern biotechnology, offering a comprehensive and high-resolution view of gene expression that enables the discovery of novel transcripts across diverse biological systems. Its applications extend beyond basic transcriptomics, providing powerful tools for uncovering molecular mechanisms underlying disease, environmental responses, and chemical toxicity. In biotechnology and biomedical research, RNA-seq facilitates the identification of regulatory networks and biomarkers that inform therapeutic development, risk assessment, and precision medicine. However, reproducibility and data interpretation challenges persist, often stemming from suboptimal experimental design or inconsistent analytical pipelines. This review critically examines key methodological aspects required for robust and biologically meaningful RNA-seq studies including experimental design, sample preparation, sequencing strategies, and data quality control focuses specifically on eukaryotic cell RNA-seq workflows. We also compare leading sequencing platforms and discuss emerging trends that enhance the scalability and reproducibility of transcriptomic analyses. By integrating best practices with recent technological advances, this review provides a practical framework for designing high-quality RNA-seq experiments that support innovation in biotechnology, systems biology, and translational research.

## Graphical Abstract

**Figure:**
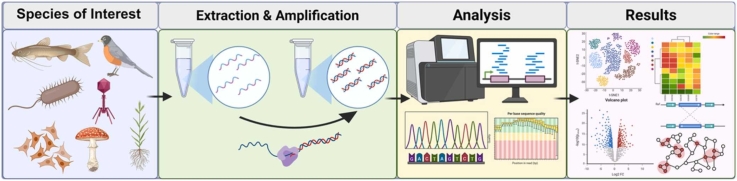

## 1 Introduction

Gene expression analysis is an inherently complex process, requiring not only technical proficiency but also a deep understanding of biological systems, data analysis, and experimental design principles. Consequently, the development of effective methodologies in this field requires close collaboration among experts from diverse disciplines, including molecular biology, biotechnology, bioinformatics, statistics, and data engineering.

Given the interconnections involved, and as research on gene expression continues to expand in both scope and frequency, it is unsurprising that the demand for interdisciplinary educational resources on gene expression studies continues to grow each year. Recent advancements in biotechnology, including CRISPR-Cas9 gene-editing technology [1] the inaugural successful utilization of mRNA vaccines in humans [2], [3] the Deep6mA software to predict DNA methylation patterns [4], the prediction of protein structures with AlphaFold [5], and the use of machine learning methods to predict the binding site of protein ligands [6], [7], underscore the rapid progression towards the accessibility of gene expression measurement and analysis tools for researchers across a broad spectrum of disciplines.

Building on these technological breakthroughs, high-throughput exploration of metabolic patterns has enabled increasingly comprehensive characterization of biological systems, including the human genome [8] proteome [9], cytome [10] and even microbiome [11]. These approaches have provided unprecedented opportunities to uncover intricate biochemical interactions, shedding light on disease mechanisms, the development of personalized medicine, and potential therapeutic interventions. Moreover, advancements in computational biology and machine learning have further refined these analyses, allowing for more precise and predictive models of human health and disease. By integrating multi-omics data, researchers can identify biomarkers, predict drug responses, and even tailor treatments to individual genetic profiles with unprecedented precision. While network-based approaches have yielded promising biomarker candidates, their clinical translation has often produced inconsistent outcomes due to challenges such as cohort heterogeneity, model overfitting, and limited reproducibility across studies, underscoring the need for rigorous validation in precision medicine [12], [13]. Altogether, gene expression analysis stands as a cornerstone methodology in modern biochemical research, offering vast applications across drug design, pharmacology, medicine, technology, agriculture, and science.

Importantly, this versatility also makes gene expression analysis a powerful tool for investigating the safety and biological impact of chemical exposures. For example, neonicotinoids, widely used as agricultural insecticides, were originally developed to maximize their affinity for insect receptors while minimizing interactions with mammalian receptors. However, until recently, no conclusive research had addressed the extent to which these compounds can interact with human receptors or influence neural function. By means of RNA-seq, it has been demonstrated that human neurons are functionally affected by low, micromolar concentrations of neonicotinoids, including acetamiprid, imidacloprid, clothianidin, and thiacloprid. The study indicates that neonicotinoids may pose a risk of developmental neurotoxicity [14], illustrating how gene expression analysis can reveal subtle but critical effects of environmental chemicals on human health.

These findings highlight a broader challenge in toxicology: while traditional assays provide essential insights into the safety of both designed drugs and environmental chemicals, they often fail to capture the full spectrum of molecular perturbations that can arise within biological systems. Traditional toxicological data, cell viability, organism-level responses, and precisely determined IC₅₀ and LD₅₀ values, remain indispensable for assessing biological risk. Yet, as highlighted earlier in the case of neonicotinoid insecticides, such conventional metrics often fall short of capturing the subtle molecular and regulatory effects that emerge at the level of gene expression. Complex biological processes, including nonlinear interactions among diverse biomolecules, cannot be fully explained without incorporating transcriptomic insights. As one critical layer of multi-omics research, transcriptomics focuses on detecting and characterizing RNA molecules central to every stage of gene regulation within the cell [15]. While early transcriptomic studies relied heavily on microarrays, the long-standing gold standard in the field, these were constrained to analyzing only known sequences, limiting the discovery of novel genes and non-coding RNAs (ncRNAs).

The advent of next-generation sequencing (NGS) overcame these limitations, with RNA-seq emerging as a powerful alternative capable of capturing a broader and more unbiased view of the transcriptome. RNA-seq enables the identification of RNA by directly reading the sequence of individual nucleotides, eliminating the need for prior binding to complementary probes, as was the case with microarrays [16]. Furthermore, transcript expression levels (after normalization and adjustment for gene length and sequencing depth) are quantified by counting reads rather than relying on signal intensity, ensuring precise and sensitive measurements largely independent of background noise. Newly discovered RNA sequences acquire full nucleotide characterization, allowing for their simultaneous identification and inclusion in databases [17]. However, the identification and annotation of new transcripts require additional bioinformatic analyses, such as de novo assembly. Although RNA-seq is not inherently targeted to a specific RNA type, the range of molecules analyzed can be narrowed depending on the research questions. For example, studies may selectively enrich for mRNA, miRNA, poly(A)-tailed transcripts, or ncRNA, thereby optimizing sequencing costs.

Currently, the rapid development of NGS technology allows researchers to choose from a variety of read lengths. Short, double-paired reads spanning 50–200 base pairs (bp) are most used. Reads of 150–200 bp are used in some applications but less frequently in routine experiments [18]. In contrast, long-read sequencing, producing reads from 1000 bp to several tens of thousands of bp, enables the identification of long non-coding RNAs (lncRNAs) and even entire viral or prokaryotic genomes [19]. Ongoing research and the continuous development of Oxford Nanopore Technology (ONT) have enabled the direct identification of long RNA strands, measuring approximately 20,000 bp, without the need to convert them to cDNA to ensure stability against ubiquitous RNAases [20].

Bulk RNA-seq, which analyzes biomolecules from thousands of cells within a tissue or organism, is often affected by contamination from other cell types or pathogens. This contamination can introduce unidentified sequences or outliers in downstream analyses. In addition, tissue-derived RNA samples are inherently heterogeneous, which complicates interpretation, for instance, through the presence of infiltrating inflammatory cells [21]. To overcome these challenges, single-cell RNA sequencing (scRNA-seq) has emerged as a powerful approach, enabling transcriptome profiling at single-cell resolution and the construction of detailed tissue maps that capture cellular diversity [22].

Transcriptomics based on RNA-seq provides powerful support for interpreting complex, multi-step, and often multidimensional interactions among biomolecules. The integration of machine learning techniques into the analysis of tens of thousands of highly correlated and significantly divergent genes enables the identification of key biomarkers [23]. However, despite its power, transcriptomics represents only one layer of the omics landscape. Because it captures a relatively limited subset of biomolecular interactions, transcriptomics alone cannot fully represent toxicity pathways or adverse outcome pathways (AOPs). For this reason, integrating multiple omics approaches, including genomics, epigenomics, proteomics, and metabolomics, is essential for a comprehensive understanding of toxicological mechanisms [24], [25].

The rapid expansion of transcriptomic RNA-seq research has generated vast datasets that continue to accumulate across diverse domains of biology, toxicology, and medicine. As these datasets continue to grow and are systematically deposited in both public repositories and proprietary platforms, their long-term scientific value increases. Researchers can now design studies with greater foresight, leveraging historical data to conduct comparative analyses, generate robust hypotheses, and even build quantitative models to validate experimental outcomes.

Sequencing methods further advance our understanding of biodiversity, evolution, and ecology, with many animal species now being characterized in unprecedented detail [26]. Highly resilient organisms, such as tardigrades and tobacco plants, can be studied to uncover the molecular basis of their extraordinary adaptive abilities, potentially driving the next wave of scientific breakthroughs [27], [28]. While detailed descriptions on performing gene expression analysis and its later exploratory data science, statistics, and clustering are quite abundant in the latest literature [29], [30] relatively little is yet published on the methodology and importance of proper experiment design. Although at first glance, planning our study might not seem like the most crucial aspect, it can make the data more reproducible for other specialist teams, especially when speaking of big data comparative studies like meta-analyses and systematic reviews. Here, project planning and management became a much more important part of scientific research, enabling a higher chance for additional conclusions drawn after the study is published.

This review aims to bridge current knowledge gaps by exploring how RNA-seq is transforming biotechnology and molecular life sciences through its capacity to deliver high-resolution, quantitative insights into gene regulation. Building upon advances that once defined the microarray era, RNA-seq now provides unprecedented opportunities to investigate molecular pathways underlying chemical toxicity, cellular adaptation, and disease mechanisms. The article offers an integrative overview of best practices for designing reproducible and biologically meaningful transcriptomic experiments, emphasizing their application in mechanistic toxicology, biotechnology, and biomedical research. Accordingly, it serves as a methodological and practical guide for scientists navigating the complexities of next-generation sequencing (NGS), with particular attention to bulk RNA-seq. We discuss key challenges in processing and interpreting large-scale transcriptomic datasets, outline critical parameters for robust experimental design, and describe essential analytical steps in differential gene expression studies. Finally, the review summarizes state-of-the-art bioinformatics tools and computational strategies tailored for eukaryotic bulk RNA-seq analysis, supporting reproducible, accurate, and mechanistically informative research that advances systems-level understanding across diverse biological systems and biotechnological applications. While the principles outlined in this review are broadly applicable across biological domains, toxicology-focused RNA-seq studies may require particular attention to dose–response relationships, temporal sampling designs, and increased biological heterogeneity, which can influence replication strategies, statistical power, and downstream analytical approaches.

## 2 RNA-seq

Compared to earlier microarray-based techniques, RNA-seq represented a major advancement by enabling unbiased transcript detection, higher sensitivity, and the ability to quantify both known and novel transcripts. Although the complexity of RNA-seq workflows remains higher, particularly with respect to sample preparation and data analysis, the benefits in terms of accuracy, resolution, and discovery power have firmly established it as the standard in transcriptomics. The most important differences between RNA-seq and microarray technologies are summarized in (Table 1 in supplementary materials).

**Fig. 1:**
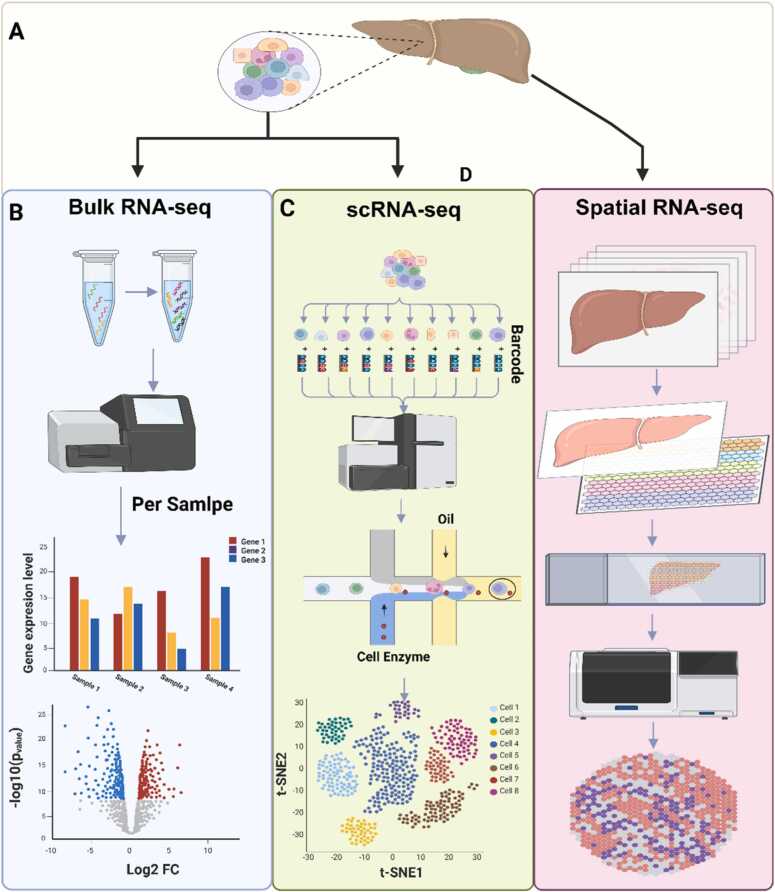
ifferences between bulk RNA-seq, scRNA-seq, and spatial RNA-seq. A a biological sample collected from the liver contains a wide range of different cell types, which can be analyzed using various sequencing techniques. B in bulk RNA-seq, all collected cells are analyzed together. After purification, reverse transcription, and cDNA synthesis, the material is directed to the sequencer, which produces averaged gene expression levels representing all cell types in the sample. C in scRNA-seq, individual cells are physically separated. Each cell is tagged with a unique barcode that enables its later identification. As each cell passes through the sequencer, it first merges with a stream of cellular enzymes and then with a stream of oil that encapsulates and isolates each cell in droplets. The resulting data reflect the gene expression profiles of individual cells. D in spatial RNA-seq, the organ is sectioned into thin slices, which are placed on slides containing unique barcoded capture probes. Cells labeled with these barcodes are then processed by the sequencer. Barcode identification allows the resulting expression data to be mapped back to the specific spatial locations within the analyzed tissue.

**Fig. 2:**
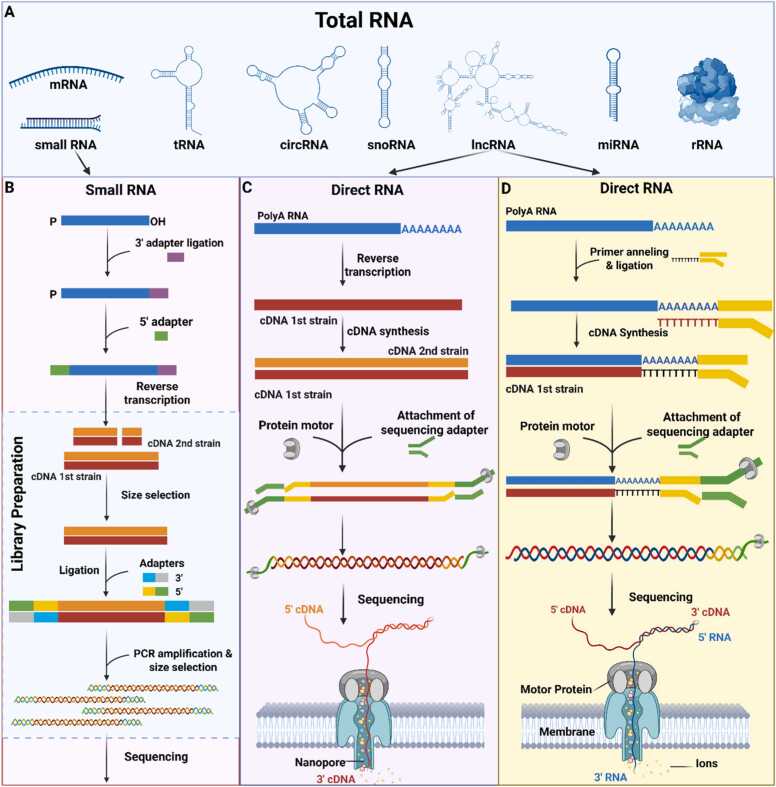
Differences in Small RNA and Direct RNA Sequencing. A Types of RNA that can be analyzed using Total RNA-seq. B At the 3′ and 5′ ends of small RNA strands, specific ligation adapters are attached, followed by reverse transcription. At this stage, library preparation takes place, during which cDNA fragments of the desired length are selected. Sequencing adapters are then added to the selected cDNA molecules, followed by PCR amplification and another size-selection step. The resulting cDNA fragments are subsequently sequenced. C In Direct RNA-seq with cDNA conversion, captured poly(A)-tailed lncRNAs undergo reverse transcription and second-strand cDNA synthesis. Sequencing adapters and a motor protein are attached to the resulting cDNA molecules, allowing the strand to pass through the nanopore during sequencing. D In Direct RNA-seq of native RNA, a primer anneals to the lncRNA molecule, initiating the synthesis of a single cDNA strand. Sequencing adapters and a motor protein are then attached to the RNA strand, which is directly sequenced without prior conversion to cDNA.

The concept of RNA sequencing is broad, and RNA-seq itself has a wide range of applications, which has led to the development of highly specialized variants designed to address specific research questions. However, this diversity also poses a challenge for beginners when selecting the most appropriate method. The most commonly used variants include bulk RNA-seq, single-cell RNA-seq (scRNA-seq), and spatial RNA-seq (spatial transcriptomics), as shown in (Fig. 1). These three platforms serve as the foundation for most types of analyses, such as direct RNA-seq, small/non-coding RNA-seq, long non-coding RNA-seq (lncRNA-seq), and tRNA-seq), as shown in (Fig. 2).

**Bulk RNA-seq** is the most widely used form of RNA sequencing, characterized by a relatively straightforward bioinformatic analysis of the obtained data. It enables a comprehensive assessment of gene expression levels in biological samples containing many cells simultaneously [15]. Results are expressed as the average activity of the entire population under study, which means that this method does not allow for the assessment of cellular heterogeneity [31]. The lack of specificity also implies that variation in sample composition, such as the presence of different cell types, may affect result interpretation, while rare cell populations and their unique expression profiles can be obscured. A major advantage of Bulk RNA-seq is its versatility: it can be applied to virtually any type of cell, including those derived from tissues, cell cultures, blood, plants, or bacteria [16]. It is essential, however, that the material is either fresh or properly preserved [32]. Because this approach does not account for heterogeneity, it is best suited for comparing gene activity under different biological conditions, such as treatment, mutation, or stress, as well as for distinguishing between healthy and diseased cells. This makes it possible to identify biomarkers associated with disease or therapeutic response [16]. An additional strength of Bulk RNA-seq is its relatively low cost, which makes it particularly useful for analyzing large patient cohorts and extensive clinical datasets. Moreover, new Bulk RNA-seq technologies based on barcoding now enable the simultaneous analysis of many samples at dramatically reduced costs up to 25 times cheaper than standard methods [33].

**scRNA-seq** overcomes the limitations of averaged gene activity measurements provided by conventional bulk RNA-seq. Cell isolates obtained from cultures or tissues are separated into individual cells using microdroplets, with each cell encapsulated in a separate oil droplet containing a unique barcode [34]. Following cell lysis and the reverse transcription of mRNA into cDNA, transcripts can be assigned to individual cells based on their unique barcodes. This fundamental approach enables single-cell resolution, uncovering cellular heterogeneity even within the same tissue [35]. This approach is particularly valuable for analyzing tumor tissues, studying embryonic development, and detecting rare cell types or transitional states. Moreover, scRNA-seq enables the reconstruction of cellular differentiation trajectories [36]. However, the individual analysis of each cell is considerably more expensive than bulk RNA-seq, making it less suitable for large cohort studies. Moreover, the resulting data are far more complex multidimensional, noisy, and non-standard. Each cell represents a separate dataset, and the number of cells in a single experiment can reach tens of thousands. scRNA-seq also requires high-quality biological material: cells must be viable, intact, and properly isolated. Dead cells can release RNA, leading to contamination, while cell aggregates complicate the assignment of transcripts to individual cells [37]. Additionally a single cell contains extremely small amounts of genetic material, of which mRNA constitutes only about 1–5 %. This quantity is insufficient to prepare an RNA-seq library directly, which is why scRNA-seq protocols require one or more amplification steps. Gene drop-out may arise both from the inherently low RNA content of the cell and from losses occurring during reverse transcription or amplification. Although any cDNA synthesis step can introduce bias in RNA-seq workflows, in scRNA-seq this effect is amplified because consecutive rounds of amplification further exacerbate it. As a result, transcript representation becomes uneven, increasing the likelihood of ”gene drop-out“ one of the major sources of artifacts in scRNA-seq analyses. In practice, this may lead to incorrect conclusions about gene expression patterns [38].

**Spatial transcriptomics (spatial RNA-seq)** is a variant of RNA-seq that enables the analysis of gene activity levels while preserving the spatial configuration of the cells that make up a tissue or organ. Unlike bulk RNA-seq and scRNA-seq, it allows for the study of cells within intact tissue sections. One method of sample preparation involves creating tissue slides that are placed on plates containing specific barcodes. The identified transcripts are then mapped to their corresponding locations on the plates, generating a topographic map of cellular activity on each slide [39]. By default, spatial RNA-seq provides two-dimensional maps, but when combined, they can be used to reconstruct a three-dimensional tissue map, distinguishing cellular activity across the entire tissue [40]. Integrating tissue morphology with transcriptomic information makes it possible to identify different types of cells, including tumor, glial, and immune cells within the context of their surrounding microenvironment. Analyzing this microenvironment provides insights into cellular interactions [41]. Mapping the developmental trajectories of organs and comparing healthy and diseased tissues is also highly valuable. Although spatial RNA-seq offers substantial benefits, the resulting data are multidimensional and complex, requiring advanced bioinformatics tools and considerable computing power. Another limitation is its relatively low resolution compared to single-cell sequencing. The 10x Genomics Visium platform typically provides a resolution of around 55 µm, capturing multiple cells per spot, which represents a significant constraint [42]. One solution to this limitation is to combine scRNA-seq with spatial transcriptomics, although this approach comes with significantly higher costs and even more complex transcriptomic data [43].

**Total RNA-seq** is the most general and comprehensive method for RNA analysis, as it enables the detection of all RNA species, including long non-coding RNAs (lncRNAs) longer than 200 nucleotides. This approach allows for the discovery of novel lncRNAs and provides valuable insights into transcriptional regulation and chromatin structure [44]. It is particularly useful in studies of neurodegenerative diseases and gene expression regulation. Transfer RNAs (tRNAs), due to their extensive chemical modifications and complex secondary structures, require specialized analytical methods. Research on tRNA modifications and their roles in translation and the oxidative stress response often involves overcoming challenges related to adapter ligation [45], [46]. Strong chemical modifications such as pseudouridylation and methylation can hinder reverse transcription and adapter ligation, making these molecules particularly difficult to analyze. Dedicated protocols such as Hydro-tRNAseq and ARM-seq have been developed to address these limitations [47], [48]. Extremely small RNAs (<18 nt) or highly structured fragments may be poorly represented or entirely undetectable using standard approaches. Therefore, studies focusing on tRNAs or small RNAs often require specialized protocols, such as small RNA-seq [49]. Ribosomal RNA (rRNA) can also be analyzed; however, because it constitutes the majority of total RNA in the cell, its presence is generally undesirable when investigating other RNA classes. Consequently, a standard step in preparing samples for Total RNA-seq involves RNA purification and enrichment through rRNA depletion.

**Clarification of terminology**: In this review, the term “bulk RNA sequencing” refers to any RNA sequencing method in which a specific RNA type (for example, mRNA) is extracted from a mixed population of cells and sequenced as a pooled sample, without the ability to resolve intercellular variability. This definition is independent of the library preparation protocol used. The term “total RNA sequencing,” on the other hand, refers to a specific library construction strategy aimed at preserving and sequencing a broad spectrum of RNA molecules, including non-polyadenylated transcripts. In this approach, various classes of RNA are sequenced after the removal of ribosomal RNA, which constitutes 80–90 % of the total RNA pool and numerically dominates over other RNA types. The depletion of rRNA enables the generation of a more diverse set of RNA molecules. In this sense, the term “total RNA” does not, by default, include the sequencing of rRNA, although rRNA sequencing from eukaryotic cells is used in specialized applications [50], [51].

**Small RNA sequencing (small RNA-seq**) enables the identification and quantitative analysis of small non-coding RNA molecules ranging from 18 to 35 nucleotides in length. Specific ligation adapters are attached to both ends of the small RNA, followed by reverse transcription. After size selection, sequencing adapters are added to the selected cDNA molecules, followed by PCR amplification and another size-selection step. This technology opens new avenues for understanding gene regulation, disease mechanisms, and cellular defense responses. Unlike traditional RNA sequencing, which focuses primarily on mRNA, small RNA-seq targets RNA species with regulatory and protective functions. This group includes: microRNAs (miRNAs), which regulate gene expression by promoting mRNA degradation or inhibiting translation [52]; small interfering RNAs (siRNAs), involved in cellular defense mechanisms such as antiviral responses [53]; piwi-interacting RNAs (piRNAs), which silence transposons in germ cells and influence epigenetic regulation [54]; tRNA-derived fragments (tRFs), originating from tRNA, which have regulatory functions and are active under cellular stress [55]; and, snRNAs/snoRNAs, which participate in splicing and rRNA modifications. Due to their typical length (60–300 nt), the latter are not always fully captured by small RNA-seq protocols [56]. This approach is highly effective for discovering novel small RNA species, including isoforms and precursors. However, it also presents several challenges. Many miRNAs differ by only 1–2 nucleotides (isomiRs) [56], making precise identification difficult. Moreover, adapter ligation bias may favor certain sequences, and some miRNAs are inherently difficult to detect due to their secondary structure or chemical modifications [57], [58], [59]. These factors can lead to errors in quantification and functional interpretation [60]. Despite these limitations, small RNA-seq remains an invaluable tool for exploring the complex molecular landscape of the cell.

**Direct RNA-seq (dRNA-seq)** is a sequencing technology that enables the direct reading of RNA sequences in their native form, as well as after RNA-to-cDNA conversion during reverse transcription. Because RNA is highly unstable and rapidly degraded by ubiquitous RNases, direct analysis was long considered impossible. Earlier approaches, such as bulk RNA-seq or scRNA-seq, relied on reverse transcription to convert RNA into more stable cDNA, which was then sequenced. In dRNA-seq, developed by ONT, a helicase motor protein binds to an RNA strand that has adapters pre-attached to both ends. The motor protein translocates the RNA through nanopores, small protein channels embedded in a membrane in the 3′ (poly-A) to 5′ direction [5]. As each nucleotide passes through a nanopore, it produces a characteristic change in the electrical current, which is detected and assigned to the corresponding nucleotide using machine learning algorithms. This approach eliminates errors introduced during reverse transcription and amplification [61], [62], [63]. Nanopore sequencing enables the reading of thousands of nucleotides in a single run, allowing for the coverage of full-length genes [64]. It also facilitates the identification of transcript isoforms, quantification of poly-A tail lengths, and detection of epitranscriptomic modifications such as methylation [65]. Signal shifts can be used to identify not only canonical nucleotides but also numerous chemical modifications, although such analyses are not yet standardized in routine laboratory practice. dRNA-seq is characterized by lower accuracy compared to traditional Illumina platforms. Moreover, since data are generated in the non-standard FAST5 format, bioinformatic analysis is more complex and requires substantial computational resources and specialized software**.**

Conducting RNA-seq studies correctly involves more than merely analyzing the raw data generated by the sequencer. A critical component is thorough experimental planning, starting with defining the study’s objectives, whether it involves profiling mRNA, investigating the entire transcriptome, or focusing on specific RNA species such as small RNAs or long non-coding RNA. It also involves determining the number of samples and measurements needed to detect the expected changes, selecting the sequencing platform, and addressing subsequent steps such as quality control of reads, preprocessing, mapping or *de novo* assembly, aligning reads to the reference genome, normalization, and differential analysis [66], [67].

Beyond data generation, RNA-seq studies involve a series of computational steps. The extensive process of analyzing data derived from NGS has led to the development of a vast array of bioinformatics tools. These tools either support individual steps, such as normalization or read mapping, or enable comprehensive analysis workflows. This accessibility allows researchers without advanced bioinformatics training to independently analyze their results. Unfortunately, this convenience is often accompanied by the improper selection of methods or parameters within applications, which can result in inaccurate outcomes [18], [67].

## 3 RNA-seq experiment design

Conducting a reliable RNA-Seq experiment is more challenging and demanding than performing a typical microarray analysis. Every decision made during the experimental design stage can determine whether the obtained data will be suitable for verifying the proposed research hypothesis. It is crucial to ensure that the generated data are of high quality and can be reused in other analyses, rather than being limited to a single study due to poor experimental planning.

Given the growing role of designing in modern science, reproducibility should be considered a key priority, as it ensures the reusability of data for testing different hypotheses. Such an approach not only benefits the scientific community but also increases the visibility and citation potential of published results. As mentioned earlier, gene expression analysis is a complex methodology that brings together experts from multiple disciplines, capable of interpreting and translating scientific findings in diverse ways. Therefore, understanding and properly setting research planning priorities can be transformative for both science and industry.

**Fig. 3:**
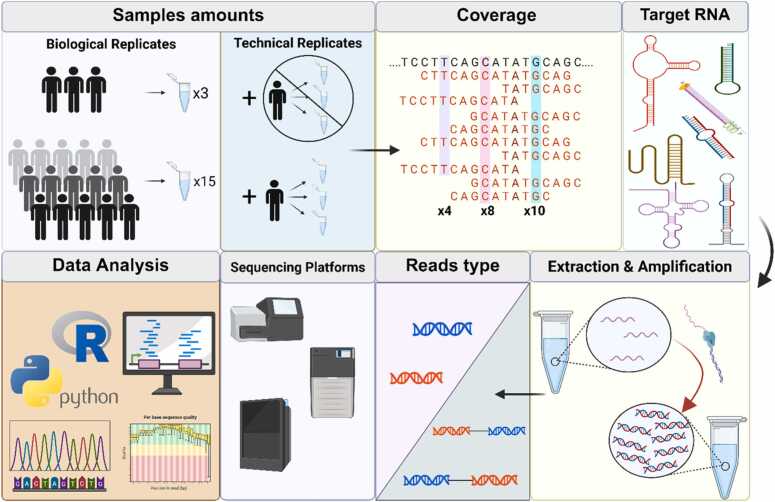
Individual steps to take when planning an experiment. A simplified diagram illustrating the key steps involved in planning an RNA-seq experiment. The sequence of blocks, indicated by arrows, reflects the order in which each step is discussed later in this chapter.

This article outlines the key aspects of experimental design that ensure reproducible results (Fig. 3) in bulk RNA-seq studies of eukaryotes, which currently represent the most prevalent application of next-generation sequencing. Proper planning extends beyond maintaining internal consistency. It also enables meaningful comparisons with other datasets, supports integration into large-scale meta-analyses, and facilitates adaptation to evolving transcriptomic standards. Adhering to reproducible research practices including the use of standardized protocols, comprehensive metadata annotation, and open data sharing can substantially increase the long-term scientific value and impact of RNA-seq datasets across diverse research domains.

### 3.1 Sample amounts for biological and technical replicates

RNA-seq is characterized by much higher reproducibility of measurements compared to microarrays [68]. Considerable variability can occur between experiments prepared using the same protocol, which is why at least two technical replicates should be included. Technical replicates are rarely used, as greater emphasis is placed on biological replicates. This is because technical reproducibility is typically well controlled by the experimental protocol. Although in higher eukaryotes, such as humans and mice, the use of technical replicates is fully justified for accurately detecting allelic imbalances or subtle changes in gene expression, studies on prokaryotes are generally characterized by well-controlled technical variability. In these cases, most of the observed variation arises from biological sources, which underscores the importance of including biological replicates in experimental design [69].

A similar situation is observed in fungi. *Saccharomyces cerevisiae* shows relatively low interindividual variability, which allows for a reduction in the number of biological replicates under controlled laboratory conditions. Transcriptomic analyses have revealed only limited variability in UTR regions across different *S. cerevisiae* strains grown under homogeneous conditions [70]. In contrast, pathogenic species such as *Candida auris* display much greater phenotypic and transcriptional variability, leading to differences in gene expression even among strains with similar sensitivity to antifungal agents [71]. Comparative analyses of *S. cerevisiae* populations have further shown that differences in promoter regions and regulatory mutations contribute to changes in gene activity, indicating high population-level variability [72]. Nevertheless, in the case of clinical isolates, substantial transcriptional heterogeneity suggests that biological replicates are far more critical than technical replicates [71]. Nevertheless, few RNA-seq studies incorporate technical replicates, even though they are essential for distinguishing biological variability from technical noise [73]. It must be strongly emphasized that in the absence of biological replicates, no population-level inference can be made. As a result, any p-value calculations are invalid and cannot be used in further analysis [18].

Moreover, previous studies have shown that using only three biological replicates enables the detection of merely 20 %–40 % of statistically significant genes. In contrast, employing more than 20 replicates allows for the identification of over 85 % of significantly differentially expressed genes (SDEs), regardless of fold change. It is generally recommended that RNA-seq experiments include at least six biological replicates, with the number increased to a minimum of 12 when comprehensive detection of SDE genes across all fold changes is required. When fewer than 12 replicates are available, the balance between true positives and false positives is best achieved with tools such as edgeR and DESeq2. The primary advantage of increasing the number of replicates is the enhanced sensitivity of SDE detection [74]. This number, however, should be understood as an idealized recommendation derived from power analyses rather than a prescriptive standard, and replication strategies must be adapted to the objectives and logistical limitations of each study. Furthermore, multi-run sequencing can introduce batch effects that obscure true biological differences; thus, when replication is constrained, experimental design and downstream analysis should incorporate strategies to detect and correct batch effects, such as randomization across runs and statistical adjustment.

A larger number of samples combined with deep sequencing allows for the detection of more subtle changes, but also leads to a significant increase in costs, which poses a major challenge for research teams with limited budgets. The optimal sequencing depth depends heavily on the specific objective, such as scRNA-seq or whole transcriptome analysis. The R library **SsizeRNA** [75] (Sample Size Calculation for RNA-Seq) allows for predicting the sample size used in an experiment with the target power, considering variable fold changes for specific genes, while also enabling the validation of predictions through a built-in function. **PROPER** [76] (PROspective Power Evaluation for RNAseq) provides methods to assess the relationship between power and sample size in the context of differential expression (DE) detection from RNA-seq data, taking into account the read depth. It performs a comprehensive evaluation of statistical power and the actual type I error depending on the sample size, based on simulation studies.

### 3.2 An appropriate number, length, and sequencing coverage of reads in relation to the project under consideration

The number of sequencing reads required in RNA-seq is closely linked to the scientific objectives of the study, the complexity of the target transcriptome, and the specific RNA class under investigation. Reported requirements typically range between 5 million and 200 million reads per sample, reflecting the wide diversity of experimental goals.

For general transcriptome profiling and the detection of changes in highly expressed genes, 10–30 million reads per sample are usually sufficient, with 20–30 million considered optimal [77]. More detailed analyses, such as identifying subtle expression changes, constructing gene expression profiles, or investigating alternative splicing, generally require 30–60 million reads per sample. While this range is often a practical compromise, highly complex analyses may demand deeper sequencing [78], [79], [80]. For alternative splicing studies, particularly in higher eukaryotes with large genomes, sequencing depths exceeding 60 million reads are frequently recommended [81]. Comprehensive transcriptome reconstruction or the discovery of novel transcripts may necessitate 100–200 million reads per sample.

Targeted sequencing experiments require substantially less coverage. For example, 3′ RNA-seq can often be performed with only 3 million reads per sample, while small RNA or miRNA sequencing typically requires 1–5 million reads [79], [82]. Other reports suggest that ≥ 10 million reads per sample provide improved miRNA detection [83]. The theoretical redundancy of coverage (c) is described as $c=\frac{L\times N}{G}$, where L is the read length, N is the number of reads, and G is the haploid genome length [84]. This formula underscores the relationship between sequencing depth and transcriptome complexity.

The optimal read length depends on the experimental goals, library design, and sequencing platform. For quantitative gene expression analysis, short single-end (SE) reads of 50–75 bp typically provide sufficient coverage. However, for comprehensive transcriptome characterization, including detection of low-abundance genes, isoform diversity, and transcript reconstruction, longer paired-end (PE) reads (e.g., 2 × 75 bp or 2 × 100 bp) are recommended [85]. For small RNA profiling, short reads remain effective; for instance, 50 bp SE reads typically achieve adequate coverage due to the limited size of the molecules.

Subsequent studies have confirmed that increasing read length and sequencing accuracy improves transcriptome assembly, especially when using third-generation platforms [86], [87]. Longer PE reads also enhance isoform resolution, splice junction detection, and *de novo* transcriptome assembly. Although reference-based strategies remain effective for well-annotated genomes, reliable detection of rare or novel transcripts often requires not only deeper sequencing but also additional biological replicates. Longer PE reads also enhance isoform resolution, splice junction detection, and *de novo* transcriptome assembly. Although reference-based strategies remain effective for well-annotated genomes, reliable detection of rare or novel transcripts often requires not only deeper sequencing but also additional biological replicates.

Some sequencing platforms still allow very short reads (25–36 bp), which can reduce costs. However, such short reads are rarely used in modern RNA-seq due to mapping limitations in repetitive or complex regions. A minimum read length of 50 bp has become the standard. Benchmarking experiments in which 100 bp PE reads were trimmed to simulate shorter read sets demonstrated that 50 bp PE reads perform comparably to 75 or 100 bp reads for differential expression analysis. Effective detection was achieved with PE reads ≥ 50 bp, whereas SE reads showed reduced mapping accuracy in complex genomic regions [85]. For isoform resolution, alternative splicing detection, and novel transcript discovery, longer PE reads of 75–100 bp are strongly recommended. Importantly, as sequencing costs decline, these strategies are becoming more accessible for routine transcriptomic studies.

The required sequencing depth and read length also depend on genome structure. The NCBI database lists over 4600 eukaryotic genomes, of which only 35 are complete according to NCBI standards (gapless chromosome assemblies). Among these, *Caenorhabditis elegans* is the only animal genome, while the rest are primarily fungi and protists with relatively small genomes. This highlights that most eukaryotic genomes remain incomplete. Because eukaryotic genomes are often large and highly repetitive, long-read sequencing is particularly valuable for *de novo* genome assembly [88].

Third-generation sequencing platforms have demonstrated clear advantages in this context. For example, long-read sequencing successfully assembled all 16 chromosomes of *Saccharomyces cerevisiae* into continuous sequences [89]. Such advances underscore the importance of long-read data for resolving repetitive regions, improving genome assemblies, and enhancing transcriptome completeness.

### 3.3 Double-stranded cDNA sequencing of target RNA on the RNA-seq platform

Contrary to common assumptions, RNA-seq does not directly sequence RNA molecules but instead relies on the synthesis of complementary DNA (cDNA). While RNA-seq is most often employed for quantification of mRNAs, other RNA classes can also be analyzed depending on library preparation strategies. However, mRNA is chemically and enzymatically less stable than DNA due to the presence of the 2′-hydroxyl group, which makes it more susceptible to hydrolysis [90]. This intrinsic instability, combined with the ubiquitous activity of RNases, prevents direct sequencing of native RNA under conventional workflows. Consequently, standard RNA-seq protocols rely on reverse transcription of RNA into more stable cDNA, which can be efficiently amplified by polymerase chain reaction (PCR) prior to sequencing.

The instability of RNA remains a major challenge, as RNases are widespread in biological samples and difficult to fully inactivate, leading to degradation of RNA molecules [91]. In contrast, DNases can be easily inactivated without affecting the quantity of cDNA, further underscoring the stability advantage of DNA-based approaches. Additionally, PCR-based protocols are inherently optimized for DNA templates. Given that mRNA typically constitutes only ∼5 % of total cellular RNA [92], the conversion of mRNA into cDNA is a critical step in ensuring sufficient material for sequencing. In a standard workflow, mRNA is first reverse-transcribed into cDNA, amplified by PCR, and then converted into double-stranded cDNA libraries that are sequenced.

Despite their centrality, cDNA-based RNA-seq workflows introduce limitations. Reverse transcription and PCR amplification can generate biases, including uneven transcript coverage, preferential amplification of certain sequences, and loss of information about RNA modifications. To address these issues, recent advances have focused on methods for dRNA-seq. ONT has pioneered such approaches through platforms such as MinION and GridION, which enable long-read sequencing of native RNA molecules spanning several thousand nucleotides [93]. In this method, RNA molecules, including poly(A) tails, can optionally undergo reverse transcription to stabilize the strand, producing RNA–cDNA hybrids. Importantly, only the RNA strand is sequenced, while the cDNA strand serves to improve throughput and stability. Sequencing adapters are then attached to either the RNA–cDNA hybrid or single-stranded RNA [94].

One of the key advantages of ONT’s dRNA-seq is that it eliminates the need for PCR amplification and complete cDNA synthesis, thereby reducing sample preparation time from approximately 300 min in cDNA workflows to ∼140 min in dRNA workflows [95]. Furthermore, dRNA-seq provides additional insights that are inaccessible to traditional RNA-seq, including the identification of polyadenylated transcripts, measurement of poly(A) tail length, and detection of base modifications such as methylation [61]. These capabilities allow researchers to capture a more accurate picture of the native transcriptome. However, despite growing academic interest, ONT-based dRNA-seq remains limited by relatively high error rates, with read accuracies of around 90 % reported for individual species [96], [97], [98].

It should be noted that ONT technology exhibits a substantially higher error rate, with typical read accuracy ranging around 85–90 % [99], whereas the Illumina platform is characterized by a much lower substitution error rate of approximately 0.1 %. The higher error frequency reduces the reliability of results when detecting isoforms or identifying rare variants. The elevated error rate in dRNA-seq reads makes it more difficult to unambiguously assign sequences to the correct isoforms, and with a low number of reads even single errors may lead to incorrect alignment to the reference transcript or to the erroneous identification of novel isoforms. As a consequence, weakly represented isoforms may remain undetected. In the case of variants with low allelic frequency, the high error rate in ONT may also mask true mutations or falsely suggest their presence. Therefore, dRNA-seq is not an optimal method for analyses requiring reliable detection of rare variants.

In addition to dRNA-seq, other emerging techniques aim to extend the scope of transcriptomic analyses. One example is NERD-seq (Nanopore Enrichment of RNA Data Sequencing), designed to improve the detection of small non-coding RNAs (ncRNAs), which are often overlooked or discarded in standard approaches. Its mechanism is similar to dRNA-seq but introduces a key modification: short reads (<200 nt) are separated from long reads (>200 nt) before poly(A) tails are added. Both fractions are subsequently polyadenylated, combined, and processed for sequencing. NERD-seq enables the expanded detection of diverse ncRNAs, including snoRNA, snRNA, scRNA, srpRNA, tRNA, and rRNA [100]. By broadening the repertoire of captured transcripts, this method offers significant potential for advancing knowledge of RNA biology, identifying novel small RNAs, and enriching the transcriptomic landscape. At present, however, NERD-seq remains largely confined to research applications and is not yet widely implemented in routine transcriptomic studies.

#### 3.3.1 RNA instability and degradation

Although we have discussed RNA instability in the context of cDNA synthesis, the issue is far broader and is a frequent source of misunderstandings and methodological errors that should be clarified before planning an experiment. It is therefore essential that we have emphasized the importance of this issue.

Both RNA stability, thermodynamic properties, and secondary structure have a significant impact on RNA-seq results as well as on the reproducibility of biological and technical replicates. RNA is chemically less stable than DNA (due to the presence of the 2′-OH group and its susceptibility to RNases), which leads to faster degradation in the sample and greater variability between replicates when collection or storage conditions are suboptimal. [101]. Degraded RNA samples, such as Formalin-Fixed, Paraffin-Embedded (FFPE) tissues, clinical biopsies, and ecological material, pose specific challenges for RNA-seq. RNA fragmentation results in a strong 5′–3′ coverage bias, reduced transcript integrity, and increased technical variance. Under degradation conditions, uneven transcript coverage (often loss of 5′ ends), an increase in unassigned or multimapping reads, and reduced correlation between replicates are commonly observed [102].

In practice, RNA quality metrics such as the RNA Integrity Number (RIN) perform well for fresh or frozen samples. However, it should be noted that fresh or frozen biopsies containing high-quality RNA are rarely available in clinical studies. Many clinical samples are archival FFPE specimens, in which RNA is often partially degraded. Therefore, DV200 (the percentage of fragments >200 nt) is a more predictive measure, as it correlates with the likelihood of successful library construction and overall NGS performance. In such cases, rRNA-depletion–based libraries are more suitable than polyA+ libraries [103], [104]. It is important to emphasize that RNA degradation is a universal, global, and stochastic process occurring in cells shortly after isolation from the organism. As the level of degradation increases, the number of DEG)’s also rises. Therefore, in RNA-seq analyses especially those focused on lncRNAs special caution should be taken when working with degraded biological samples [104].

Some studies indicate that longer transcripts are more susceptible to loss of coverage under RNA degradation, whereas others show that this relationship becomes weak once degradation modeling and appropriate normalization methods are applied. Therefore, a safe and reasonable strategy includes assessing RNA quality (RIN/DV200) before library preparation, selecting a protocol that is robust to degradation (such as ribo-depletion or 3′-end protocols), increasing sequencing depth, applying fragmentation-related metrics such as the Transcript Integrity Number (TIN), and incorporating a sufficient number of biological replicates to account for variability introduced by RNA degradation [102], [104]

Different classes of small and non-coding RNAs exhibit substantial variability in sequence composition, secondary structure, and chemical modifications [105]. These properties have major implications for RNA-seq experimental design. Small RNAs, such as miRNAs and piRNAs, require dedicated small-RNA-seq protocols because their short length and high sequence heterogeneity are not reliably captured by standard RNA-seq workflows [106], [107]. Other ncRNAs, including snoRNAs, snRNAs, and tRNAs, contain extensive base modifications that can inhibit reverse transcription and introduce amplification biases, leading to uneven coverage and inaccurate quantification [108], [109]. Long non-coding RNAs often show low expression, variable transcript lengths, and incomplete or absent polyadenylation, which necessitates the use of ribo-depletion-based protocols. Together, these intrinsic sequence and structural features determine the appropriate library preparation, sequencing strategy, and bioinformatic pipelines, and they directly affect the interpretability and reproducibility of RNA-seq results [110].

### 3.4 The required amount of biological material in extraction & amplification

The amount and quality of biological material used to prepare cDNA libraries are critical factors in RNA-seq experiments. This challenge is amplified by the fact that the human body contains approximately 200 distinct tissue types distributed across about 50 organs, each with unique transcriptional signatures. Cells from different tissues often exhibit distinct expression profiles, and RNA-seq may obscure this heterogeneity by averaging signals across diverse subpopulations. Preserving cellular or tissue homogeneity is therefore essential to avoid loss of biologically meaningful information [111]

The choice of material depends on the study’s objectives as well as the amount of input RNA available. Compared with microarrays, RNA-seq requires considerably less starting material, with typical input amounts ranging from 10 ng to 1 µg. A single human cell contains less than 1 pg of mRNA [112] yet advances in RNA amplification technologies now make it possible to generate libraries from single cells. By amplifying and synthesizing double-stranded cDNA, modern platforms can produce sufficient quantities of material from small samples. However, the use of extremely low input amounts is not generally recommended, as it increases the risk of amplification bias and gene drop-out, especially in scRNA-seq.

There are commercial kits available that enable complete library preparation from as little as 1 ng of input material [113]. It is important to note that each platform may have different requirements regarding the minimum amount of material needed for optimal performance. Therefore, the manufacturer's specifications for a given platform should always be consulted.

Before proceeding with library preparation, it is necessary to enrich or deplete total RNA. Total RNA includes rRNA, mRNA, pre-mRNA, miRNA, siRNA, piRNA, long non-coding RNAs, and other types of RNA. Since ribosomal RNA can constitute 80–90 % of the total RNA content in a cell, using such samples without prior depletion would result in minimal detection of mRNA, which is the target of gene expression analysis [114]. It should also be remembered that there are many specialized platforms available on the market, such as 10x Genomics, Smart-seq3, STRT-seq, for which the minimum requirements vary depending on the chosen platform and the technology used.

### 3.5 Single-end and paired-end sequencing read types

**Fig. 4:**
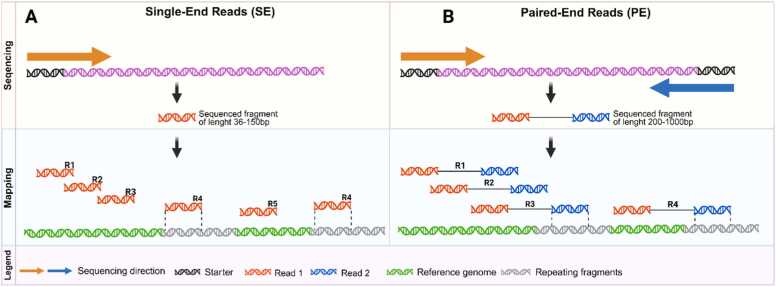
Comparison of SE read & PE read. A SE involves sequencing from only one end of the DNA strand, indicating the direction in which the sequence proceeds. This approach generates a large number of reads, which are then individually mapped to the reference genome. SE provides a cost-effective way to generate large amounts of high-quality reads. B PE involves sequencing the same DNA strand from two directions, both the 3′ and '5′ ends, allowing for high-quality data for sequence mapping. PE sequencing facilitates the detection of genomic rearrangements, repetitive elements, gene fusions, novel transcripts, as well as deletions and insertions, which are significantly more challenging to detect with SE. It is good to note that the sequencer generates one file for SE and two files for PE, corresponding to Read1 and Read2.

After reverse transcription and library preparation, the cDNA is subjected to sequencing using either SE or PE strategies. These approaches differ in read structure, mapping accuracy, and downstream applications, and the choice between them depends largely on the study design and research goals [115]. Both read types differ in nature and in the way they are subsequently mapped to the reference genome, as visualized in (Fig. 4).

**Single-end sequencing** generates reads from one end of each DNA fragment. This produces a single continuous read per fragment, which is then aligned independently to the reference genome. While computationally straightforward, SE reads can present challenges in mapping, particularly when repetitive elements exceed the read length, leading to ambiguous assignments across multiple genomic loci. **Paired-end sequencing**, in contrast, sequences both ends of a DNA fragment, generating two reads (one from each end) with a known insert size. This additional spatial information allows for more accurate mapping across repetitive or structurally complex genomic regions, providing greater confidence in alignment [16], [116]. As a result, PE sequencing typically yields more reliable estimates of transcript abundance and enhances the accuracy of differential gene expression analysis [117]. Although PE does not eliminate mapping errors in repetitive regions, the availability of fragment length information substantially increases mapping precision compared to SE sequencing.

In practice, cost and project scope strongly influence the choice between SE and PE. SE sequencing remains attractive for large-scale or population-level studies where cost efficiency is critical and where only the overall number of reads per gene is of interest [118]. It is also widely used in targeted applications such as 3′ RNA-seq (e.g., QuantSeq), where read directionality is sufficient to quantify gene expression. However, as sequencing costs continue to decline, PE sequencing is increasingly preferred, even with shorter reads, since it improves alignment quality, reduces ambiguities, and provides richer information for transcriptome assembly and isoform detection.

### 3.6 Available sequencing platforms

Currently, the market offers a wide range of commercially available RNA sequencing platforms from various manufacturers. Competition and continuous technological advancements have led to the development of specialized approaches tailored to different research needs. Some manufacturers have focused on short-read sequencing and high-throughput platform development. For example, Illumina systems allow sequencing of fragments ranging from 25 bp to 300 bp, while Ion Torrent supports reads up to ∼600 bp, although its use in RNA-seq has become less common in recent years. On the other hand, companies such as Pacific Biosciences (PacBio) and ONT have dedicated their efforts to improving long-read sequencing technologies. Both platforms can routinely generate read lengths of 10–25 kb, with maximum read lengths extending much further, thereby enabling the sequencing of full-length transcripts and isoform resolution. For instance, ONT’s MinION has enabled the sequencing of intact protein strands on commercially available nanopore sensors, achieving single-amino-acid sensitivity [119]. However, this is an experimental approach that extends nanopore technology beyond RNA and DNA. The choice of the ONT platform should be closely guided by the study’s objectives, for example, ONT is particularly valuable when obtaining epigenetic or structural information is crucial. However, it is not an optimal solution in situations where the priority is the identification of rare events with high accuracy. Although dRNA-seq enables richer biological information and direct reads, this comes at the cost of lower accuracy and reduced throughput. In contrast, Illumina technology offers high precision and sensitivity, but at the expense of losing information about RNA modifications.

The choice of platform should be guided by the research objective. It is important to keep in mind that some platforms are designed for short-read sequencing, while others are intended for long reads. It is also crucial to consider the type of sequencing technology, such as sequencing-by-synthesis (SBS), nanopore, or single-molecule real-time (SMRT), and the format in which the results will be stored. It should be noted, however, that the vast majority of studies are conducted using Illumina technology.

Depending on the sample size and research objectives, a single RNA-seq experiment can generate data ranging from several gigabytes (GB) to several terabytes (TB), classifying it as Big Data. A summary of companies offering various platforms (Table 2 in Supplementary materials), along with their products and specifications, represents only a small fraction of the commercially available options. A more comprehensive overview of sequencing platforms and their applications can be found in recent reviews [19], [120], [121]

When choosing a sequencing platform, it's important to understand which type of error predominates, as this has implications for variant analysis or de novo assembly. Substitutions occur when the sequencer misreads a nucleotide for example, recording a G instead of an A. This is typical of Illumina technology (short-read, SBS), which is characterized by a very low error rate. Substitutions are usually easy to correct with high coverage. Indels refer to errors involving the deletion or insertion of nucleotides. These are common in ONT technology and older versions of PacBio. Indels are more difficult to correct than substitutions, especially in homopolymeric regions containing long stretches of the same nucleotide, such as “AAAA”.

Different sequencing platforms rely on distinct physical units in which the sequencing reaction occurs. In general, Illumina and ONT platforms use Flow Cells, whereas PacBio systems use SMRT Cells, and these units differ in structure and sequencing principles. A Flow Cell refers to a nanopore-containing cartridge in ONT platforms or a surface for DNA cluster generation in Illumina systems. In contrast, a SMRT Cell used in PacBio systems contains thousands of Zero Mode Waveguides (ZMWs), [122] with each ZMW enabling real-time observation of a single polymerase synthesizing DNA. These platform-specific units define the capacity, throughput, and experimental flexibility of each sequencing technology

### 3.7 Python or R: Which programming language is better for data analysis?

The computational ecosystem surrounding RNA-seq analysis comprises a broad range of tools that differ in both functionality and programming requirements. Many of the most widely adopted frameworks for differential expression analysis, including DESeq2 115, edgeR, [113], [114] and limma [123], are implemented in R and therefore require basic knowledge of the R programming environment, particularly data manipulation, statistical modeling, and visualization. In contrast, tools for high-dimensional exploratory analysis and workflow automation, such as PyDESeq2 [116] are predominantly written in Python, and their effective use benefits from familiarity with Python syntax, package management, and data structures. Python-based workflows are also frequently chosen for projects that integrate RNA-seq with machine learning, spatial transcriptomics, or large-scale multi-omics analysis because of the extensive ecosystem of scientific computing and numerical libraries.

Beyond proficiency in a specific scripting language, the Unix command line has become a fundamental skill for modern RNA-seq analysis. Many key tools for quality control, trimming, alignment, quantification, and file handling, such as FastQC, fastp, Trim Galore, STAR, HISAT2, samtools, and bedtools, are executed primarily through Unix-based shells rather than graphical interfaces. As a result, even analysts working primarily in R or Python must often combine scripting with command-line execution to develop efficient, reproducible workflows. This is particularly relevant in high-performance computing environments, where analysis is distributed across cluster nodes using job schedulers and pipeline managers. Familiarity with Unix-based environments also facilitates reproducibility, as command scripts can be version-controlled, containerized, and automatically executed.

For researchers without prior programming experience, a number of code-free or graphical platforms provide accessible alternatives that allow users to run RNA-seq workflows without writing scripts. Popular solutions include the Galaxy platform [112], which provides a browser-based interface to a large collection of tools, and graphical implementations of RNAlysis[111], which integrate exploratory analysis, clustering, and visualization. Such platforms lower the barrier to entry for experimental biologists and can be effective for small-scale or exploratory work. However, they also introduce notable limitations. These tools are generally less flexible and harder to customize, particularly when users need to deviate from standard workflows, incorporate new methods, or troubleshoot pipeline failures. In addition, graphical environments tend to be less efficient for large-scale processing, may lack full transparency of parameter settings, and often require manual interaction, which can reduce reproducibility and hinder automation. For these reasons, command-line and scripting-based workflows remain the preferred approach for large datasets, high-throughput studies, and projects requiring scalability, reproducibility, and integration of multiple analysis steps.

Overall, rather than advocating for a single “best” language, the practical question is how researchers can most effectively combine R, Python, and Unix-based tools to build workflows that are reproducible, scalable, and appropriate for their experimental goals. The diverse and complementary ecosystems surrounding these tools reflect the evolving nature of RNA-seq analysis and underscore the importance of computational literacy in modern transcriptomics.

#### 3.7.1 Batch effects

Batch effects arise when samples are processed in different “batches,” that is, groups that differ in at least one technical aspect of sample handling or library preparation. Batch effects are one of the major sources of unwanted technical variability in RNA-seq datasets. If samples are not evenly balanced across batches, they can severely distort biological interpretation. Such effects may result from differences in sample handling, RNA extraction batches, library preparation kits, sequencing lanes or flow-cells, RNA degradation levels, or even differences between personnel and storage conditions. Because RNA-seq is a highly sensitive technique, even small inconsistencies during sample preparation can introduce substantial technical variability. To ensure reliable data interpretation, batch effects must first be detected and, if present, properly corrected [124].

Since experiments are rarely perfect, Principal Component Analysis (PCA), Multidimensional Scaling (MDS), clustering, and correlation analyses should be standard components of every RNA-seq pipeline. These exploratory tools allow researchers to diagnose issues such as RNA degradation, outlier libraries, lane or flow-cell biases, or inconsistently processed samples, often enabling early intervention or exclusion of problematic data.

The most common strategy for detecting batch effects is exploratory data analysis. Principal Component Analysis (PCA) is a widely used dimensionality-reduction method. If samples cluster according to a technical parameter rather than a biological condition, this indicates the presence of a batch effect. Multidimensional Scaling (MDS) visualizes similarities or distances between samples, making technical batches appear as distinct clusters. Hierarchical clustering can further reveal grouping driven by parameters such as extraction date or laboratory personnel. Another useful approach is sample-to-sample correlation matrices, which rely on the observation that samples from the same batch often show artificially high correlation with each other and lower correlation with samples from other batches patterns that frequently reflect technical artifacts, poor RNA quality, or library preparation errors [125], [126], [127].

Before performing differential expression analysis, several well-established and widely described statistical methods should be applied to reduce batch effects. **ComBat/ComBat-seq** provides parametric or non-parametric empirical Bayes correction [128]. **RUVSeq** estimates and removes unwanted variation using control genes or replicate samples [127]. Limma’s ***RemoveBatchEffect*** performs linear correction after data normalization [129]. **Mixed-model approaches** such as including batch as a fixed or random factor in DESeq2 or edgeR models are another robust strategy [130], [131]. **SeqQscorer** is a machine learning tool that estimates the probability of low quality for a given RNA-seq, DNAse-Seq, or ChIP-Seq sample [126]. These methods reduce technical noise while preserving the true biological signal. A key aspect of proper batch correction is to avoid applying it automatically; exploratory analyses should first confirm that the chosen method restores clustering according to biological conditions rather than technical batches.

## 4 Data formats

A critical component of modern RNA-seq analysis is the use of workflow management systems that enable scalable, reproducible, and automated execution of bioinformatic pipelines. Tools such as Nextflow [132] facilitate modular pipeline design, transparent parameter specification, and efficient parallelization across local workstations, HPC clusters, or cloud environments. This framework helps reduce human error, track dependencies, and ensure that identical outputs can be reproduced from the same inputs, even when run on different computational infrastructures. In parallel, version control systems such as Git (often combined with GitHub, GitLab or Bitbucket) support good software engineering practices by allowing users to track changes to scripts, collaborate asynchronously, perform code review, and maintain a history of pipeline versions [133].

**Fig. 5:**
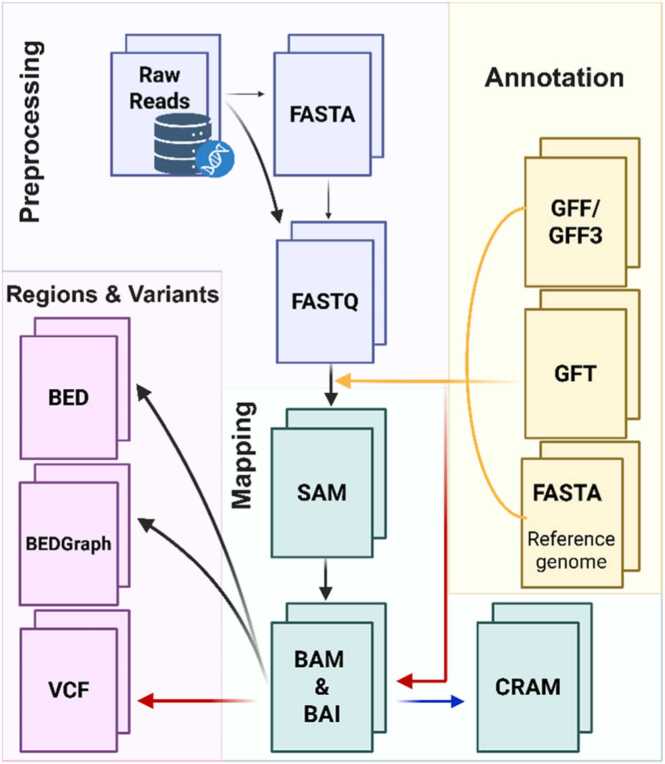
Data format Roadmap. The Data Format Roadmap has been simplified to enhance its readability. Each of the presented data formats can be converted into another; for example, SAM can be transformed into BAM, and similarly, BAM into SAM. However, marking the arrows to indicate bidirectional conversion could introduce unnecessary clutter to the diagram and make it less intuitive for a first-time encounter with the topic. The arrows shown illustrate the steps of transcriptomic data analysis, from data acquisition to obtaining the required formats for subsequent stages. The most commonly used bioinformatics tools and libraries for format conversion can be found in the subsection. Typically, raw data from the sequencer was delivered to the user in FASTA format. The FASTA file was then converted to FASTQ format, which additionally included quality scores. However, increasingly, sequencer output is delivered in the vast majority of cases in FASTQ format. This makes converting FASTA to FASTQ unnecessary, but still possible in specific cases. To map sequences into SAM format, an additional FASTA file with the reference genome and annotation files is required. After successful mapping, the SAM file can be converted into its compressed version, BAM + BAI (both files must be present together), and subsequently into an even more compressed format, CRAM. Unlike BED and BEDGraph formats, the VCF format also requires annotation files.

The actual analysis of RNA-seq is as diverse as how it can be applied, which also means that various file formats are encountered at different stages of the workflow. The following section outlines the actions included in each step (Fig. 5), how they are performed, and which file formats are required.

### 4.1 Preprocessing

The raw data generated by the sequencer are by default saved in a FASTQ file, which contains the nucleotide sequences (reads) for each sample enriched with quality scores for each base call has been the currently standard. Although it is technically possible to convert a FASTA file into a FASTQ file, this operation is neither standard nor recommended in RNA‑seq workflows. The quality information contained in FASTQ files cannot be recovered once lost, because FASTA files do not store per‑base quality scores. Therefore, performing such a conversion is artificial and justified only in limited situations, such as when analyzing historical datasets that lack quality information. In those cases, quality scores are generated artificially by assigning a uniform value or random values; however, such approaches are not reliable for quantitative or qualitative analyses. For these reasons, converting FASTA to FASTQ is generally discouraged. An exception to this rule is Oxford Nanopore Technology, whose output files are typically in FAST4 or FAST5 format. If SE sequencing was performed, each sample is associated with one FASTQ file. In the case of PE sequencing, there are two FASTQ files, one for each end. Quality assessment of each sample is a mandatory step before proceeding with further analysis. Even with carefully planned experiments, artifacts may occur and must be identified and addressed at this stage.

Quality assessment includes evaluating metrics such as per-base and per-sequence quality, nucleotide content per sequence, GC content, presence of unidentified bases, read length distribution, sequence duplication levels, overrepresented sequences, and adapter contamination. Tools like **FastQC** are free and widely used for assessing read quality, especially from Illumina platforms. In contrast, **NGSQC** [134] can be used across various sequencing platforms. FastQC generates both text and HTML reports that visualize the aforementioned statistics. One of the most important metrics is per-base quality, which reflects the average quality of the sequenced reads. In general, high-throughput sequencing data should show consistently high per-base quality scores across most read positions, while only a small fraction of bases or reads should fall into lower-quality ranges. Lower scores in the terminal regions are common and do not necessarily disqualify reads from downstream analysis. A detailed description of quality assessment and interpretation can be found in the (sub-section 6 “Quality Scores”) supplementary materials.

Base composition per sequence should be balanced in DNA sequencing, with lines representing each nucleotide running roughly in parallel. In RNA-seq, deviations from this pattern may appear in the first 10–15 nucleotides due to biological and technical factors. The GC content should follow a normal distribution. In RNA-seq, however, deviations (either higher or lower peaks) from the theoretical distribution may occur. A shifted normal distribution indicates systematic bias unrelated to base position. A warning is triggered if 15 % of reads deviate from a normal distribution, while a 30 % deviation is flagged as a failed metric. Read length distribution should ideally center around the expected length (e.g., 75 bp), though trimming may alter this distribution. The presence of unidentified bases indicates positions where the sequencer failed to accurately determine the nucleotide. Ideally, such bases should be absent. In RNA-seq, duplicate reads from highly abundant transcripts are expected and do not indicate technical error. Detection of overrepresented sequences may signal either biological relevance or library contamination, prompting the need for deduplication. If such sequences are found, FastQC will list potential sources of overrepresentation. Frequently, adapters appear as overrepresented sequences or attach to the 3’ end during library construction, necessitating their removal.

If reads show low per-base quality (e.g., < Q25), trimming the low-quality ends is recommended. Additionally, reads that are too short (e.g., < 20 nucleotides) should be removed. Tools like **FASTX-Toolkit** [135], **Trimmomatic**, **Cutadapt**, and **fastp** [136] can be used for these preprocessing steps. Although **Fastp** [137] is not as well-known or widely used as **Cutadapt**, it offers full deduplication functionality, enabling simultaneous trimming and deduplication. Other available tools for adapter trimming include **Flexbar** [138] and **BBMap** [139]. For trimming low-quality bases, tools such as **FASTX-Toolkit**, **PrinSeq** [140], and **SolexaQA** [141] can be used. To remove very short reads, **PrinSeq** and **Trim Galore** [142] they are also suitable.

One of the most commonly used and popular tools is **Trimmomatic**, which can handle all three preprocessing tasks: trimming, deduplication, and adapter removal. Trimmomatic includes two modes: one for **SE** and another for **PE** data. It's important to note that trimming PE reads must be performed on both reads simultaneously. If one end is discarded due to insufficient length, the remaining read becomes unpaired and will be treated as SE. A good practice is to **reanalyze read quality** after trimming, deduplication, and adapter removal.

In some cases, however, trimming of low-quality bases is intentionally skipped. This is because mapping tools such as **STAR** ^143^ are equipped with **soft clipping mechanisms**, which can handle low-quality bases at the ends of reads automatically. When working with large-scale data, it is advisable to use **MultiQC** [144], which generates an HTML report that **summarizes quality metrics across all samples** in a single, easy-to-navigate file. Bioinformatic processing must also account for degradation by employing integrity metrics like TIN, using quantification methods robust to uneven coverage (like Salmon or Kallisto) and avoiding analyses sensitive to transcript completeness, such as isoform reconstruction. Together, these adjustments are essential to obtain reliable expression estimates from low-quality or degraded RNA.

### 4.2 Annotation

To align previously prepared reads to a reference genome, the **Genome Index** must first be created. A Genome Index is a specialized data structure that enables rapid and efficient alignment of sequencing reads by indexing the reference genome. Without this structure, aligning reads linearly across the entire nucleotide sequence would be extremely time-consuming and inefficient. Annotation files for specific organisms can be downloaded from publicly available databases such as **GENCODE** [145], **UCSC Genome Browser** [146], **Ensembl** [147], and **NCBI RefSeq** [148]. From these databases, one should download the nucleotide sequence of the genome of interest in **FASTA format**, along with gene annotation files in **.gtf** or **.gff** format, which include the structure of genes and exons. Gene Transfer Format (GTF) and General Feature Format (GFF) are widely used file formats in bioinformatics for storing annotation data. GFF is extensively employed for representing complex genomic data, making it valuable in genome mapping, annotation, and comparative genomics. It provides users with a detailed overview of genomic features in DNA, RNA, and proteins. On the other hand, GTF specializes in the representation of genes and their structures, which is especially crucial in analyzing gene expression within a cell. This format facilitates gene mapping and quantification of expression levels. Both formats play integral roles in various bioinformatics pipelines, tailored to their respective strengths and use cases. Both file formats can be converted. **AGAT** [149] is a free bioinformatics tool in Python that provides scripts for manipulating and analyzing genomic annotation files. It allows for the conversion between GTF and GFF formats, making it a valuable tool for users needing to switch between these formats in genomic data workflows. **GenomeTools** is a set of bioinformatics tools in Python that includes numerous functions for analyzing genomic data. It also allows files to be converted between GFF formats, enabling users to seamlessly switch between GFF and its variations [150].

#### 4.2.1 Annotation source & Genome indexing

Despite appearances, genome indexing based on available annotation sources may seem like one of the less significant technical steps, but in reality it is an important and often underestimated issue. Annotation resources are continuously updated, supplemented with previously missing information, and corrected when errors are identified. Different databases apply distinct methodological approaches to selecting and assigning annotations, which means that results obtained from two different sources can differ substantially. This has critical implications both for experimental design and for assessing reproducibility. Ensuring the ability to replicate previous analyses is particularly important therefore, any re-indexing of the genome should always rely on the same version of the annotations that was used by the authors of the original study.

Building a Genome Index is time-consuming; however, it only needs to be done once for a given reference genome. Several specialized tools are available for this task, including: **HISAT2** [151], **STAR** [143], **Bowtie2** [152], **TopHat2** [153], **Salmon** [154], **Kallisto** [155]. It is crucial to perform genome indexing using the same tool that will later be used for alignment. Equally important is ensuring consistency in the version of the reference genome and the annotation file. Interestingly, the source from which annotation files are obtained can significantly impact downstream quantification results. For instance, using human annotations from RefSeq has been shown to yield better quantification outcomes compared to the more comprehensive annotations from Ensembl. However, the latest updates to RefSeq have resulted in decreased annotation accuracy. This highlights how the choice of annotation source and version influences subsequent results [156].

### 4.3 Mapping

RNA-seq generates millions of reads per sample that need to be mapped before quantification can be performed. Classic tools like BLAST work well for searching small numbers of sequences but are not efficient enough for mapping such large volumes of reads across the entire genome in a reasonable amount of time. To address this problem, tools for mapping, such as STAR, TopHat, HISAT2, **BWA** [157], **Minimap2** [158], **Bowtie, Trinity** [159] were proposed and are currently widely used, while more tools can be found here [160]. Mapping involves assigning reads to their corresponding locations in the genome or transcript. This task becomes particularly challenging when the user has very short reads, which, due to their insufficient length, may be mapped to multiple regions. This can limit the use of longer PE reads. It is important for the selected tool to be "splice-aware," meaning it should be capable of aligning reads that contain exon sequences from two exons on either side of an intron. Examples of such tools include: STAR, HISAT2, **GSNAP** [161], **SOAPSplice** [162].

The presence of a reference genome determines whether the mapping will be performed using the reference genome or whether a more complex genome assembly will be required. If the user has a reference genome, mapping can be performed either to the genome or to the transcriptome. Reads can be mapped uniquely to the genome or may have multiple potential alignment sites (multireads), which occur due to the presence of repetitive sequences in the genome. Mapping to the transcriptome will result in an even higher number of multireads, as the reads are aligned to all gene isoforms [18].

Mapping to the genome with gap mapping during the protocol takes into account gaps such as introns that separate the coding sequences. The result of this mapping can be carried out in two ways: by incorporating annotation that appropriately identifies transcripts, or without annotation, which allows for the discovery of new transcripts that must then be functionally annotated, for example, using **Blast2GO** [163]. Mapping to the transcriptome is performed without considering introns. This approach also does not allow for the identification of new transcripts. A third approach is mapping without a reference genome through *de novo* assembly, using dedicated tools like **Trinity**, **SOAPdenovo-Trans** [164], **Oases** [165], **Trans-ABySS** [166] or **StringTie**[167], [168]. In this approach, longer PE reads are preferred, as they provide much more informative data than SE reads. Additionally, short reads do not allow for the identification of the exact start and end positions of a given transcript [159]. Initially, the reads are assembled into contigs or transcripts. These same reads are then mapped onto the *de novo* assembled reference genome, followed by functional annotation. The results obtained from successful mapping are stored in.sam,.bam, or.cram formats, which can later be converted into.bed and.vcf formats to store information about mapped regions and variants. After alignment, reads are assigned to genomic features to generate a count matrix for downstream differential expression analysis. This can be accomplished using traditional count-based tools, such as FeatureCounts [169], which efficiently summarize reads overlapping annotated genomic features, or using probabilistic transcript quantification approaches, such as Salmon [170], which estimate transcript abundance by modeling read uncertainty, sequence bias, and multi-mapping effects.

Incomplete or poorly annotated transcript regions, together with the natural structural heterogeneity of RNA molecules, can introduce coverage biases that impair read mappability, quantification accuracy, and downstream interpretation. Missing segments or highly structured regions may lead to local mapping dropouts or uneven representation during fragmentation and reverse transcription. To minimize these effects, experimental design should consider the completeness and quality of the reference and, when appropriate, incorporate strategies such as longer reads, long-read sequencing, or transcriptome assembly, while analytical workflows should account for structure-dependent and reference-related biases to ensure robust RNA-seq interpretation.

An essential yet sometimes overlooked aspect of RNA-seq data analysis is the visual inspection of aligned reads using genome browsers, such as IGV (Integrative Viewer), UCSC Genome Browser, or Ensembl Browser. These tools enable researchers to view genomic sequences along with many types of annotation data, providing insights that may be difficult to detect solely through numerical metrics [171]. Genome browsers are particularly valuable for validating unexpected results, identifying technical artifacts (e.g., misaligned reads, rRNA contamination, or fragmented transcripts), and confirming biologically relevant features such as alternative splicing or allele-specific expression [172]. Because visualization integrates quantitative signals with genomic context, it serves as a critical step for quality control, method validation, and interpretation, complementing statistical approaches and helping researchers assess whether computational outputs reflect underlying biology rather than technical bias.

## 5 Data bases

**Fig. 6:**
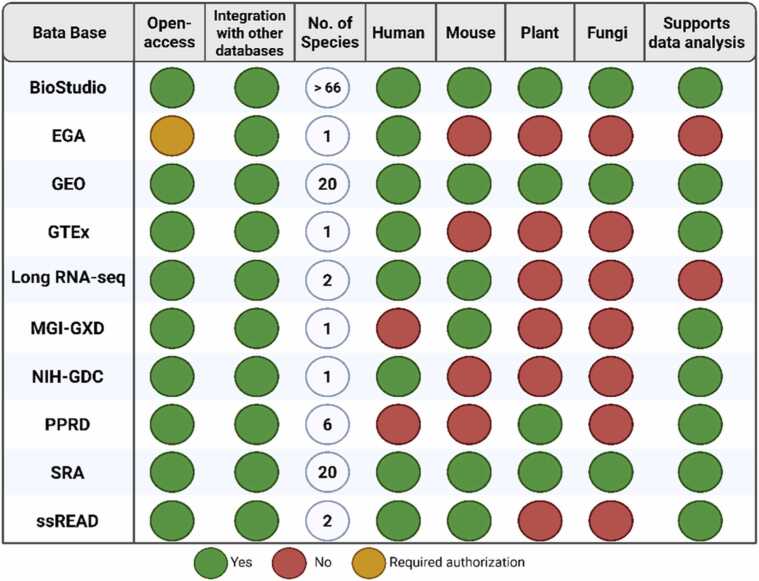
Comparison of Data Base for RNA-seq. The columns Human, Mouse, Plant, and Fungi indicate whether a given database contains sequencing data derived from these organisms.

The advancement of medicine, biotechnology, and pharmacy has driven growing interest in microbiological studies and cell-based tests, which continuously generate vast amounts of transcriptomic data. The long-term value of this data increases as it is systematically stored in public and proprietary databases. Over the past decade, a significant shift in dominant analytical platforms has occurred. In 2013, RNA-seq accounted for approximately 13 % of expression data, while nearly 67 % came from microarrays. By 2022, RNA-seq transcripts represented 70 % of the data deposited in the **Gene Expression Omnibus (GEO**) [173], [174], whereas microarrays contributed less than 10 % [175]. The massive influx of terabytes of data from various organisms has led to the creation of numerous repositories that aggregate new datasets. These resources enable researchers to plan experiments more effectively, leveraging comparative preliminary studies and quantitative models to validate hypotheses based on past research. Available databases vary in scope, containing data from multiple species or focusing on a single organism, allowing for diverse dataset selection and reanalysis as detailed in (Fig. 6).

**BioStudies** [176], [177] is a vast database that includes descriptions of biological studies sourced from other databases, thereby facilitating access to them. It contains data from the largest number of organisms compared to other databases. One of the integrated databases is **ArrayExpress** [178], [179], [180], [181], [182] which is connected to The European Bioinformatics Institute (EMBL-EBI), which contains a functional genomics data collection and stores data from high-throughput functional genomics experiments. Additionally, it allows users to download experimental data in FASTQ format and raw data from the European Nucleotide Archive (ENA) website. Expression Atlas [182], [183], like BioStudies, is connected to EMBL-EBI. Expression Atlas is an open science resource that gives users a powerful way to find information about gene and protein expression. Its catalog includes 66 species, including plants and fungi. Users can also view visualizations and download differential and baseline experiments. **Sequence Read Archive (SRA)** [184], [185] is a repository directly linked to GEO, containing all raw data for all datasets in GEO. Files containing > 5 GB must be downloaded using the SRA Toolkit. **ssREAD** [186] is the smallest repository mentioned above, but it contains only transcriptomic data for Alzheimer’s disease patients. **ReCount3** [187], [188] is an extension and continuation of the Recount project, which offers easy access to processed and normalized RNA-seq data, such as RNA-seq gene, exon, and exon-exon junction counts from multiple public repositories. Recount3 also provides a set of tools for reprocessing RNA-seq data, allowing uniform analysis from different sources without the need to manually download and process the data. The data is normalized and prepared to be ready for further analysis, such as differential gene expression analysis (DE).

**The European Genome-Phenome Archive (EGA)** [189] is a secure database designed for the permanent archiving and sharing of personally identifiable genetic and phenotypic data resulting from biomedical research projects. It hosts a wide variety of datasets, including those generated from RNA-seq experiments, whole-genome sequencing (WGS), and epigenetic studies. Researchers can access the data through an authorization process that safeguards sensitive information while supporting large-scale genomic and transcriptomic research across human populations. **Genotype-Tissue Expression Project (GTEx)** [190] is a major NIH-funded initiative aimed at understanding how genetic variation influences gene expression across different human tissues. The database provides comprehensive RNA-seq profiles from thousands of tissue samples collected from diverse individuals. GTEx serves as an essential resource for studying tissue-specific gene regulation, expression quantitative trait loci (eQTLs), and mechanisms underlying human disease susceptibility. The open-access data can be used for integrative analyses linking genomics, transcriptomics, and clinical phenotypes. **Long Read RNA-Seq** [191] represents a collection of standardized datasets and processing pipelines developed as part of the Encyclopedia of DNA Elements (ENCODE) project to address challenges associated with long-read RNA sequencing technologies, such as those produced by PacBio and ONT. These data provide high-resolution insights into transcript isoforms, alternative splicing, and full-length transcript reconstruction, complementing traditional short-read RNA-seq. The ENCODE pipeline ensures consistent processing and annotation, making it a key resource for researchers studying transcriptome complexity and gene regulation. **Mouse Genome Informatics - Mouse Gene Expression Database (MGI-GXD)** [192] is a comprehensive resource for gene expression information in the laboratory mouse, integrating data from diverse experimental platforms, including RNA-seq, in situ hybridization, and immunohistochemistry. The database allows users to explore temporal and spatial patterns of gene expression across different tissues, developmental stages, and experimental conditions. MGI-GXD is an integral consortium, supporting translational research and comparative genomics by providing high-quality, curated data relevant to human disease models. **NIH Genomic Data Commons Data Portal (NIH -GDC)** [193] is an extensive data-sharing platform developed by the National Cancer Institute (NCI) to provide unified access to genomic and clinical data from cancer studies, including RNA-seq datasets. The portal integrates data from large-scale projects such as The Cancer Genome Atlas (TCGA) and Therapeutically Applicable Research to Generate Effective Treatments (TARGET), offering standardized formats and powerful analytical tools for reanalysis and visualization. The GDC promotes reproducibility and collaboration in cancer research by enabling users to access, process, and harmonize multi-omic data within a single ecosystem. **Plant Public RNA-Seq Database (PPRD)** [194] is a curated and integrative database dedicated to plant transcriptomic research. It compiles publicly available RNA-seq data from a wide range of plant species, reanalyzed using a consistent bioinformatics pipeline to ensure comparability across studies. PPRD provides visualization tools for differential gene expression, co-expression networks, and functional annotations. This platform facilitates large-scale meta-analyses in plant biology, enabling insights into gene function, environmental responses, and evolutionary processes.

## 6 Conclusions

The rapid diversification and continuous refinement of RNA-seq methodologies are providing unprecedented opportunities to generate accurate, comprehensive, and large-scale transcriptomic datasets. Parallel advances in bioinformatics, still a relatively young but fast-evolving discipline, have produced a vast ecosystem of tools for RNA-seq data analysis. While this diversity enables sophisticated insights, it can also appear overwhelming to newcomers entering the field.

In this review, we sought to highlight the major benefits of RNA-seq for biological research, with a particular emphasis on its application in differential gene expression analysis. By combining a literature overview with practical guidance on experimental planning and sequencing platform selection, we aimed to provide experimentalists with a structured entry point into RNA-seq workflows. Although our focus was on gene expression profiling, many of the principles and practices discussed here are broadly applicable to other RNA-seq applications, including alternative splicing, isoform detection, small RNA profiling, and de novo transcriptome assembly. In addition, beyond traditional differential expression, network-based approaches such as Weighted Gene Co-expression Network Analysis (WGCNA) are increasingly applied to RNA-seq data to identify co-regulated gene modules, reveal systems-level relationships, and support interpretation of complex biological phenotypes, particularly in longitudinal or multi-factorial studies.

It is important to stress that the analysis of RNA-seq data is equally as critical as the experimental design phase. Handling such datasets requires not only statistical and computational expertise but also access to adequate computational infrastructure, as data processing can be resource-intensive and time-consuming. Nevertheless, with careful planning and informed use of available tools, RNA-seq continues to empower researchers to uncover complex biological processes, identify biomarkers, and contribute to advances in precision medicine, agriculture, and biotechnology.

Finally, we emphasize that this review captures only a fraction of the rapidly expanding knowledge base in the field. Readers are strongly encouraged to consult supplementary materials, specialized reviews, and continually updated bioinformatics resources to remain abreast of methodological innovations and best practices. As sequencing technologies and computational methods continue to evolve, RNA-seq will remain a cornerstone of modern biological research, driving discovery across multiple scientific domains.

## Funding

This work was funded via the National Science Centre in the frame of the TransNANO project (UMO-2020/37/B/ST5/01894).

## Author statement

We, the authors, declare that the revised manuscript is original, approved by all authors, not under consideration elsewhere, and that all authors meet authorship criteria. We disclose that we have no conflicts of interest.

## CRediT authorship contribution statement

**Tomasz Puzyn:** Writing – review & editing, Project administration, Funding acquisition. **Karolina Jagiello:** Writing – review & editing, Writing – original draft, Validation, Supervision, Conceptualization. **Klaudia Chmielewska:** Writing – review & editing, Writing – original draft, Methodology, Investigation. **Kamil Antoszewski:** Writing – original draft, Visualization, Software, Resources, Methodology, Investigation, Formal analysis, Data curation.

## Declaration of Competing Interest

The authors declare that they have no known competing financial interests or personal relationships that could have appeared to influence the work reported in this paper.

## Data Availability

This article is a literature-based systematic overview and does not contain original research data. All data supporting the findings are extracted from published articles and publicly available databases, which are properly cited in the manuscript. No new datasets were generated. Additional information can be provided by the corresponding author upon reasonable request.

## Footnotes

Appendix A Supplementary data associated with this article can be found in the online version at doi:10.1016/j.csbj.2025.12.015.
