# Supplementary material for: Designing RNA sequencing experiments: A practical guide to reproducible gene expression analysis
Source: Comput Struct Biotechnol J. 2025 Dec 18;31:101–19. doi: 10.1016/j.csbj.2025.12.015 (PMC12795997; doi:10.1016/j.csbj.2025.12.015)
Supplement: Supplementary file 1 — Supplementary material [file mmc1.docx]

**Supplementary Materials**

# **Dynamic Range**

Table 1 Comparison of RNA-seq and microarray characteristics

| **Feature** | **Microarray** | **RNA seq** |
| --- | --- | --- |
| **Principles** | Hybridization-based method | Sequencing-based method |
| **Dynamic range** | ~ 3.6 *10^3^ | ~2.5 *10^5^ |
| **High run-to-run reproducibility** | No | Yes |
| **Need reference genome** | Yes | No |
| **Re-analyzable data** | No | Yes |
| **Can detect unknown genes?** | No | Yes |
| **Can detect novel alterations?** | No | Yes |
| **Can detect alternative splicing?** | No | Yes |
| **Well-defined protocols** | Yes | No |
| **Needed input** | 200 ng to 100 ng | 10 ng to 1 µg |
| **Resolution** | From several to 100 bp | Single base |
| **Throughput** | High | High |
| **Background noise** | High | Low |
| **Cost for mapping transcriptomes of large genomes** | High | Low |

Table 1 legend: Fundamental methodological and analytical differences between hybridization- and sequencing-based transcriptomic approaches. RNA-seq offers broader dynamic range, single-base resolution, and the capacity to detect novel transcripts, splice variants, and unknown genes, whereas microarrays are limited by probe design and background noise but benefit from established, standardized protocols. Values for dynamic range and input quantity represent approximate experimental ranges commonly reported in transcriptomic studies.

- 1. **Dynamic Range**

In gene expression studies, dynamic range refers to the span of signal values that a given technique can reliably detect, from the lowest, e.g., weakly expressed transcripts to the highest, highly transcribed genes. For example, transcript profiling in activated T cells demonstrated that RNA-seq has a much broader dynamic range (2.5 × 10⁵) compared to microarrays (3.6 × 10³). This broader range allows for more accurate detection of differences in gene expression, making it possible to identify both over- and under-expressed genes that may remain undetected by microarrays.

The difference arises from methodological limitations: in microarrays, the dynamic range is constrained by fluorescence signal saturation (upper limit) and background noise (lower limit), whereas in RNA-seq, the lower limit is defined by sequencing background and the number of reads per transcript, with no true upper limit other than the total number of reads obtained. As a result, RNA-seq provides better identification of rare transcripts and a more accurate characterization of genes expressed at widely varying levels ^1^.

The differences in dynamic range between microarrays and RNA-seq arise from the fundamental specifications and operating principles of both platforms. Microarrays rely on predefined target transcript sequences of specific lengths, making the physical detection of genes that were not previously included on the array impossible. In contrast, RNA-seq reads sequences in real-time, meaning there is no predefined limit to the sequences it can capture. A compelling example is the analysis of differential transcript expression in anterior cruciate ligament (ACL) tissues based on the time since injury. For example a total of 24 samples were analyzed using microarrays; 8 samples were analyzed with RNA-seq, and an additional 8 samples from the same set were analyzed with RNA-seq alongside a second microarray. RNA-seq identified 3,993 unique genes with significant expression changes, while the 24-sample microarray detected only 21 genes, and while 8-sample microarray identified 86 genes. This illustrates that, despite utilizing a sample set three times smaller, RNA-seq enables the detection of nearly 190 times more genes than microarrays ^2^.

1. **Available Sequencing Platforms**Table 2 Available Sequencing Platforms

| **Platform** | **Model** | **Unit** | **Max Reads length** | **Read type** | **Generated amount of data^a^** | **Run Time** | **Q30^b^** | **File Format** | **Primary error type** | **Reference** |
| --- | --- | --- | --- | --- | --- | --- | --- | --- | --- | --- |
| **Illumina** | HiScanSQ | Lane | 1x35bp to 2x100bp | SR & PE | 23Gb-150Gb | 36hr- 204hr (8.5day) | >80% | FASTQ | Substitutions | ^3^ |
|  | MiSeq v3 | Lane | 1x75bp to 2x300bp | SR & PE | 3.3Gb-15Gb | 21hr-56hr | >70% | FASTQ | Substitutions | ^4^ |
| **ION Torrent** | Ion Proton System | Chip | Up to 200bp | SR | Up to 10Gb | 2hr-4hr | No Data | FASTQ, SFF, BAM, and VCF | Indels | ^5^ |
|  | GeneStudio S5 Ion 530 | Chip | 200bp to 600bp | SR | 1.5Gb-8Gb | 4hr-21.5hr | >99% | FASTQ, SFF, BAM, and VCF | Indels | ^6^ |
| **Pacific Biosciences** | Onso | Lane | 1x200bp to 2x150bp | SR & PE | 80Gb-15Gb | 32hr-48hr | ≥90% (Q40+) | FASTQ | Substitutions | ^7^ |
|  | Revio | SMRT Cell | 15000bp to 20000bp | SR | 35Gb-120Gb | 12hr-30hr | 85% | BAM | Indels | ^8^ |
| **Oxford Nanopore** | PromethION 24/48 12 chemistry | Flow cell | typically 6-20 Kbp | SR | 277Gb-13.3Tb | 72hr | No Data | FAST5 | Indels | ^9^ |
| **Complete Genomics** | DNBSEQ-G400 FCS | Lane | 1x100bp to 2x300bp | SR & PE | 55Gb-180Gb | 13-98hr | >80% | FASTQ | Substitutions | ^10^ |
|  | DNBSEQ-T7 | Lane | 2x100bp to 2x150bp | PE | 1Tb-7Tb | 16hr-24hr | >85% | FASTQ | Substitutions | ^11^ |
| **Singular Genomics** | G4 - F2 | Lane | 2x50bp to 2x150bp | SR & PE | 15Gb-50Gb | 11hr-19hr | >85% | FASTQ | Substitutions | ^12^ |
| **Ultima Genomics** | UG 100 | Chip | 225bp to 300bp+ | SR | 1.8Tb-2.4Tb | 12hr-14hr | >85% | FASTQ | Indels | ^13^ |
| **Element Biosciences** | AVITI | Flow Cell | 2x75bp to 2x300bp | PE | 150Gb-180Gb | 24hr-60hr | >85% | FASTQ | Substitutions | ^14^ |

Table 2 legend:
**^a^** This is the maximum output of a full instrument run. This number is based on the manufacturer’s specifications and optimizations**.**

**^b^** Performance metrics may be impacted by application, sample quality, library preparation, loading concentration, and other sequencing considerations. Metrics are generated based on reference bacterial and human genomes.

*Lane*: A physical partition in a flow cell where sequencing reactions occur. Each lane can process a separate sample or replicate; *Chip*: A semiconductor device used in platforms like Ion Torrent that contains wells for DNA/RNA sequencing reactions; *SMRT Cell*: Single Molecule Real-Time Cell contains thousands of zero-mode waveguides for real-time single-molecule sequencing; *Flow Cell:* glass slide with nanochannels where DNA fragments bind and are amplified for sequencing; *Wafer*: substrate containing an array of nanopores used for high-throughput sequencing. It may hold multiple flow cells; *PE*: Paired-End read; *SE*: Single-End read; *Substitutions*: base is incorrectly identified (e.g., reading an A instead of a G); *Indels:* Insertions and deletions of bases are mistakenly added or removed from the sequence.

# **Data Formats**

## **FASTA:**

The **FASTA format** is a text-based format containing sequences of nucleic acids (DNA or RNA) or proteins, represented by single-letter abbreviations for nucleotides or amino acids, respectively. The first line of a FASTA file begins with the ">" character, where the first word serves as the sequence identifier, and the subsequent text provides an optional description. The second line (which can wrap) contains the sequence as a string of characters.
FASTA was developed in the mid-1980s by William R. Pearson and David J. as a result of their work on fast searching of nucleotide and protein sequences. The ultimate goal was to enable the comparison of sequences in large biological databases, such as GenBank and UniProt. The FASTA format allowed for relatively fast identification of homology and similarities between sequences, which proved very useful in protein function prediction and evolutionary analysis ^16^.

Given the purpose of the FASTA format, each file should ideally be unique and pertain to only one sequence. However, in practice, deviations from this rule occur. In various databases, it can happen that two different protein or gene sequences are recorded under identical FASTA headers. This arises from inconsistent data management across sources. Furthermore, some FASTA files may represent entire families of genes or proteins. Sequences representing different variants or isoforms of the same protein or gene may be described in separate FASTA files but may feature headers suggesting their relation.

## **FASTQ:**

A FASTQ file contains both the biological sequences represented as single-letter characters and their corresponding quality scores, encoded using ASCII characters. The **first line** starts with an "@" symbol and includes the sequence identifier and an optional description. The **second line** holds the sequence, similar to a FASTA file. The **third line** begins with a "+" symbol and may include an optional description (often a copy of the description from the first line). The **fourth line** encodes the quality scores for the sequence from the second line, with each score represented by a single ASCII character. The number of characters in this line must match the number of bases in the sequence.

## **FAST4 and FAST5:**

FAST4 format is derived from FASTQ and stores separate probability scores for each of the four nucleotide bases (A, T, C, G). **FAST5**: This is an advanced variant of FAST4 and is the standard output format for sequencing devices by Oxford Nanopore Technologies, such as the PromethION. FAST5 uses the hierarchical data format (HDF5), **which supports storing large and complex datasets.** The HDF5 format and its hierarchical structure refer to the file itself, as well as to large and complex datasets. FASTA4 and FASTA5 are generated for use by ONT (Oxford Nanopore Technology), where nucleotide identification in the sequence is based on measuring changes in voltage caused by different nucleotides passing through nanopores. This complexity is reflected in the data format. The file sizes are enormous because ONT analyzes long, and sometimes very long, reads, ranging from several thousand base pairs and beyond. Unlike FASTA and FASTQ files, FAST5 files are binary and cannot be opened with standard text editors ^17^.

## **SAM:**

The Sequence Alignment Map (SAM) file is a text-based format used for storing biological sequences aligned to a reference sequence. However, it can also store unaligned nucleotide sequence data in certain cases

A SAM file consists of a header containing metadata, such as the SAM version, followed by a row for each read in the dataset. Each row includes 11 tab-delimited fields that describe the corresponding read. Using such a solution allows for obtaining valuable information about the location of the mapped read, its quality, and the presence of variations such as deletions or insertions. A detailed description and an explanation of the meaning of individual columns can be found in the documentation^18^. Below, I will outline the most important information.

1:497:R:-272+13M17D4113chr110033863768M1S67M87N70M=1003387115CGGGTCTAGA;44999;499<8<

1. **QNAME** – Corresponds to the read name from the input FASTQ file.
2. **FLAG** – The bitwise FLAG indicates whether the read is marked as aligned, a PCR duplicate, or if its mate is mapped.
3. **RNAME** – Reference sequence name, e.g., *Chr1*, which indicates chromosome 1.
4. **POS** – 1-based leftmost mapping position.
5. **MAPQ** – Mapping quality reports how well the read has aligned to the reference and is related to its “uniqueness.” Aligners characterize their confidence in the point of origin by reporting a mapping quality score: a non-negative integer *Q = -10 log10*, where *p* is an estimate of the probability that the alignment does not correspond to the read's true point of origin. The higher the MAPQ value, the better; however, a value of 225 means that the quality is not available. Some tools for generating VCF files may ignore **MAPQ ≤ 10**, which corresponds to a **1/10 chance** that the indicated read actually originates from another location. Therefore, a cutoff threshold of **MAPQ = 10** can be applied ^19^.
6. **CIGAR** – (Concise Idiosyncratic Gapped Alignment Report) is a simplified and compact format for representing detected variations or matches within a given read. For example, the string *68M1S67M15M87N70M* means: 68 bases matching the reference, 1 soft-clipped read followed by 67 matches, 15 matches followed by 87 bases skipped, followed by 70 matches.
7. **RNEXT** is analogous to field 3 (Reference Name) and follows the same rules, except that it describes the paired-end mate of the read (if there is one). To save space, this value will be “=” if it is identical to the Reference Name value, which should be the case most often.
8. **PNEXT** is analogous to field 4 (Position) and follows the same rules as that field.
9. **TLEN** indicates the length of template sequence to which the read maps (this field is sometimes confused for the read length, which it is not, but will often be equal to in value). A read with multiple insertions may have a smaller template length than the read length, while a read with multiple deletions may have a template length longer than the read length. It is set as 0 for a single-segment template or when the information is unavailable.
10. **SEQ** (Sequence) is the actual read sequence. Should generally follow the sequence line from the source FASTQ. This field can be a "*" when the sequence is not stored.
11. **QUAL** (Quality) should generally follow the quality string from the source FASTQ file and be Phred-scaled base error probability. This field can be a "*" when quality is not stored^18^.

We might ask ourselves why a SAM file is capable of storing both mapped and unmapped reads if, in downstream analysis, we only use the fragments that we have identified on the reference genome with reasonable quality ^20^. We cannot analyze differences in gene expression levels for genes that do not exist. The answer to this question lies in the information we can extract from them. A significant percentage of reads remain unmapped and are therefore discarded in further stages of analysis. However, these very reads can reveal important biological insights and previously unseen host–pathogen relationships. The analysis of unmapped DNA and RNA reads from the great tit (*Parus major*) allowed the identification of sequence similarities to related bird species and the presence of known pathogenic species of *Parus major*. Moreover, RNA contigs belonging to other birds, animals, plants, bacteria, fungi, and viruses were also detected. A more detailed analysis revealed the identity and abundance of pathogenic organisms, gene gaps, and contamination in the reference genome ^21^. Metatranscriptomic analysis of unmapped reads from over 400 human disease studies revealed the presence of reads belonging to microorganisms and viruses ^22^. Other examples of analyses or the application of a different approach to unmapped reads are thoroughly described here ^23^.

## **BAM & BAI:**

**BAM (Binary Alignment Map)** is a compressed version of the **SAM (Sequence Alignment Map)** format, containing the same information but stored in a binary format. This makes BAM files significantly smaller in size and faster to read compared to SAM files. However, since BAM files are in a binary format, they are not human-readable and cannot be opened as plain text files. To access the information within a BAM file, it must first be converted back to the SAM format. In addition, BAM files are often accompanied by a **BAI file** (BAM index), which acts as an external index table. This index enables rapid navigation to specific regions within the BAM file without the need to read the entire file.

Nevertheless, the BAI file is only functional when used alongside its corresponding BAM file without the BAM file, it serves no purpose ^18^.

## **CRAM:**

**CRAM (Compressed Reference-oriented Alignment Map)** was established in 2011 by the 1000 Genomes consortium as a standard for storing genomic data. The continuous decline in sequencing costs per base, combined with the increasing throughput of sequencers, has made its usefulness in diagnosing, treating, and preventing both human and animal diseases increasingly recognized. Currently, genetic laboratories face the challenge of processing multiple gigabytes of data weekly. Storing such large volumes of genomic data involves ongoing costs that laboratories must bear. The solution to this problem was the introduction of the CRAM format, which requires significantly less disk space than its original BAM format version ^24^. CRAM is a columnar compressed version of the BAM format, typically 30% to 60% smaller than BAM files. It achieves this efficiency by leveraging reference-based compression and optimized data storage structures. CRAM is fully compatible with BAM, making it a **viable alternative for reducing data storage requirements while maintaining usability** ^25^. **Samtools** is a suite of free programs designed for handling high-throughput sequencing data. It supports reading, writing, editing, indexing, and viewing files in **SAM**, **BAM**, and **CRAM** formats. This versatility makes Samtools an essential tool in genomic data analysis pipelines ^26^.

## **BED:**

**BED (Browser Extensible Data)** is a format commonly used in bioinformatics to store information about genomic regions and their properties. Like GFF and GTF, BED is an interval-based format used for storing coordinates of regions in the genome. This format typically contains between 3 to 12 columns. The first three columns are mandatory, while the remaining columns are optional. The "*Chrom*" column contains the name of the chromosome or scaffold, while the "ChromStart" and "ChromEnd" columns represent the start and end coordinates of the region, respectively. There are various extensions to this format, differing in the number of columns, such as ".bed4", ".bed9", and ".bed11".

## **BEDGraph:**

BEDGraph is an extension of the BED format used to store numerical values assigned to specific genomic regions. It is commonly used to represent continuous data, such as signal intensity (e.g., from ChIP-seq, RNA-seq experiments, or coverage density data). BEDGraph is similar to the BED format but contains only four columns and has several other differences shown in (Table 3). These columns include: **Chrom**: The name of the chromosome or scaffold. **ChromStart**: The start coordinate of the region. **ChromEnd**: The end coordinate of the region. **Value**: The numerical value assigned to that genomic region (e.g., signal intensity or coverage) ^27^. One of the best tools for genomic analysis tasks is **BEDTools** ^28^, which uses simple mathematical operations, such as addition, subtraction, multiplication, and division, for genomic computations. It allows users to intersect, merge, count, complement, and shuffle genomic intervals from multiple file formats, such as BAM, BED, GFF/GTF, and VCF ^29^.

Tabela 3 Differences between BED and BEDGraph format:

| **Feature** | **BED** | **BEDGraph** |
| --- | --- | --- |
| **Content** | Genomic regions | Regions with assigned values |
| **Columns** | >3 | Exactly 4 |
| **Application** | Genomic annotations | Representation of continuous signals |

## **VCF:**

Variant Call Format (VCF) is a text file that contains information about genetic variants in a gene or DNA sequence, describing single-nucleotide variants (SNVs), insertions, deletions, and other changes in the sequence. The VCF file may also include information about the genotypes of samples for each position ^30^.

**VCFTools** ^31^ It allows performing operations on VCF files, such as comparing files, summarizing variants, validating and merging files, creating intersections and subsets of variants, filtering specific variants, and converting to various file formats ^32^.

# **Quality Scores**

When reviewing the specifications of various sequencing platforms, and especially when analyzing FASTQ files, one may encounter Quality Scores expressed as numerical values derived from Phred quality scores. Originally, the Phred scale was developed during the Human Genome Project to represent the quality of DNA sequence reads generated by sequencers. Today, it is widely used in sequencing to encode the confidence level of each base call within FASTQ files ^33,34^.

Quality Score is a numerical value that describes the precision with which a nucleotide base is identified in a recorded sequence. It represents the probability of an error in base calling and is expressed on the logarithmic Phred scale. Given an assertion, *X*, the quality score, Q(*X*), expresses the probability that A is not true, P(~*X*), according to the relationship:

$$Q\left( X \right)=-10\times\log_{10} (P(\sim X))$$

Where P(~*X*) is the estimated probability of an assertion *X* being wrong. The quality scores are logarithmically linked to error probabilities, as shown in Table 4:

Tabela 4 Quality score

| **Quality Score** | **Error probability P(~*X*)** | **Accuracy of base call** |
| --- | --- | --- |
| Q10 | 0.1 | 90% |
| Q20 | 0.01 | 99% |
| Q30 | 0.001 | 99.9% |
| Q40 | 0.0001 | 99.99% |
| Q50 | 0.00001 | 99.999% |

Table 4 legend:
Phred scores are generally assumed to range from 0 to 40; however, because they represent the probability of a correct base call, they can theoretically range from 0 to infinity. A higher Phred score indicates a greater likelihood of correctly identifying the true nucleotide, while a lower score reflects a higher probability of error. Phred quality scores can be interpreted in two ways: either as the probability of a correct base call or as the likelihood of an incorrect one. For example, a Q-score of 30 means that the nucleotide has been identified with 99.9% accuracy, corresponding to an error probability of 0.1% (since 1/1000 = 0.1%). Higher Q-scores indicate better data quality, providing a concise and standardized representation of minimal error probabilities. This metric is crucial in RNA-seq data analysis as it affects the accuracy of read mapping to the reference genome and the subsequent interpretation of biological results.

The most used cutoff value for high-quality RNA-Seq data is Q30, meaning that the probability of an error in a nucleotide base is less than 0.1%. Reads with excessively high error rates are discarded during preprocessing as part of quality control (QC) assessment. Typically, sequences are trimmed to remove fragments with Q<30. Poor quality can lead to misassignment of reads or their complete loss. If low-quality data are included in the analysis, the results may contain falsely detected differences in gene expression. A Quality Score value assigned to a specific platform, such as Q30 for 80% (as shown in Table 4), indicates that more than 80% of the nucleotides have a Q-score of 30, corresponding to a 99.9% probability of correct base identification ^35^.

Programs such as **FastQC** ^35^, **Trimmomatic** ^36^, and **Cutadapt** ^37^ analyze Quality Scores and help filter out low-quality reads. Some programs apply a cutoff threshold for bases with a quality score below Q20. Including low-quality data in the analysis can result in inaccurately detected gene expression differences, which may, in turn, lead to the misidentification of genetic variants. It is also important to remember that different platforms may have various methods for generating and evaluating Quality Scores, as they are designed for other analytical purposes. Depending on the technology, these values may vary in terms of accuracy and the way they are recorded in FASTQ files, as shown in Table 5.

Tabela 5 Quality score summary for individual platforms

| **Platform** | **Read Type** | **Typical Q-score** | **Accuracy (%)** | **Application** |
| --- | --- | --- | --- | --- |
| **Illumina** | Short (75-300 bp) | Q30–Q40 | 99.9% | RNA-Seq, SNP calling, gene expression analysis |
| **PacBio HiFi** | Long (10 kb–30 kb) | Q20–Q40 | 99%–99.99% | Iso-Seq, structural variant detection |
| **ONT** | Long (1 kb–100 kb) | Q10–Q20 | 90–98% | Full transcripts, viral RNA, single-cell analysis |

Table 5 legend:
 *^a^SNP Calling*: Detection of single-nucleotide polymorphisms (SNPs) in expressed regions of the genome by aligning RNA-seq reads to a reference genome and identifying base variations;
*^b^Iso-Seq:* Full-length isoform sequencing that accurately identifies transcript variants, alternative splicing, and 5'/3' UTR boundaries;
*^c^Structural Variant Detection*: Using long-read or high-depth RNA-seq data to identify large genomic rearrangements (e.g., insertions, deletions, translocations) that affect transcription;
*^d^Viral RNA*: RNA-seq applied to detect and quantify viral transcripts, understand host–virus interactions, or track viral genome variation;
*^e^Single-Cell Analysis:* Application of RNA-seq at the single-cell level (scRNA-seq) to dissect cell heterogeneity, identify cell types/states, and map lineage trajectories.

Overall, Illumina appears to be the best platform to use for short-read RNA-sequencing experiments. Meanwhile, ONT and PacBio are more suitable for full transcript reconstruction, such as identifying alternative isoforms. Both in the context of the sequencing quality achieved by a given platform (Table 5) and for each base in a sequence recorded in a FASTQ file (a detailed description of the FASTQ file format is provided below).

# **References**

1. Zhao, S., Fung-Leung, W. P., Bittner, A., Ngo, K. & Liu, X. Comparison of RNA-Seq and microarray in transcriptome profiling of activated T cells. *PLoS One* **9**, (2014).

2. Rai, M. F., Tycksen, E. D., Sandell, L. J. & Brophy, R. H. Advantages of RNA-seq compared to RNA microarrays for transcriptome profiling of anterior cruciate ligament tears. *Journal of Orthopaedic Research* **36**, 484–497 (2018).

3. *Illumina HiScan Specification Sheet:* https://www.illumina.com/documents/products/datasheets/datasheet_hiscansq.pdf (2010).

4. Illumina MiSeq Specification Sheet: https://www.illumina.com/content/dam/illumina/gcs/assembled-assets/marketing-literature/miseq-system-data-sheet-m-gl-00006/miseq-data-sheet-m-gl-00006.pdf (2011).

5. *The Ion Proton ^TM^ System Specification Sheet:* https://tools.thermofisher.cn/content/sfs/brochures/CO06326_Proton_Spec_Sheet_FHR.pdf (2012).

6. Ion GeneStudio S5 series Specification Sheet: https://assets.thermofisher.com/TFS-Assets/CSD/Specification-Sheets/PG1720-PJT2769-COL22253-P-on-GeneStudio-S5-Spec-Sheet-Global-FLR.pdf (2018).

7. *PacBio Onso Specification Sheet:* https://www.pacb.com/wp-content/uploads/Onso-brochure.pdf (2023).

8. Biosciences, P. *PacBio Revio Specification Sheet:* https://www.pacb.com/wp-content/uploads/Revio-specification-sheet.pdf (2022).

9. *Oxford Nanopore PromethION 24/48 Specification Sheet:* https://nanoporetech.com/products/sequence/promethion-24-48 (2019).

10. Complete Genomics DNBSEQ-G400. https://www.completegenomics.com/dnbseq-technology/ (2022).

11. Complete Genomics DNBSEQ-T7. https://www.completegenomics.com/products/sequencing-platforms/dnbseq-t7/ (2019).

12. Singular Genomics G4. https://www.singulargenomics.com/g4x (2022).

13. *Ultima Genomics UG 100 Specification Sheet*. https://cdn.sanity.io/files/l7780ks7/production/d82b05dc0310d76cb66974d11ee93ceab8664c84.pdf (2024).

14. Element Biosciences AVITI Specification Sheet. https://go.elementbiosciences.com/aviti-specs (2022).

15. bugaco.com. Fasta to Fastq Sequence Converter. https://sequenceconversion.bugaco.com/converter/biology/sequences/fasta_to_fastq.php (2000).

16. Lipman, D. J. & Pearson, W. R. Rapid and Sensitive Protein Similarity Searches. *Science (1979)* **227**, 1435–1441 (1985).

17. Oxford Nanopore Technologies. Ont fast5 api. https://github.com/nanoporetech/ont_fast5_api (2017).

18. *Sequence Alignment/Map Format Specification*. https://samtools.github.io/hts-specs/SAMv1.pdf (2024).

19. Johns Hopkins University. Bowtie2 Manual. vol. 35 https://bowtie-bio.sourceforge.net/bowtie2/manual.shtml#mapping-quality-higher-more-unique (2024).

20. Peng, X. *et al.* Re-alignment of the unmapped reads with base quality score. *BMC Bioinformatics* **16**, (2015).

21. Laine, V. N., Gossmann, T. I., Van Oers, K., Visser, M. E. & Groenen, M. A. M. Exploring the unmapped DNA and RNA reads in a songbird genome. *BMC Genomics* **20**, (2019).

22. Simon, L. M. *et al.* MetaMap: An atlas of metatranscriptomic reads in human disease-related RNA-seq data. *Gigascience* **7**, (2018).

23. Gurgul, A. *et al.* Another lesson from unmapped reads: in-depth analysis of RNA-Seq reads from various horse tissues. *J Appl Genet* **63**, 571–581 (2022).

24. Stephens, Z. D. *et al.* Big data: Astronomical or genomical? *PLoS Biol* **13**, (2015).

25. samtools-devel. *CRAM Format Specification (Version 3.1)*. vol. 21 https://samtools.github.io/hts-specs/CRAMv3.pdf (2025).

26. Petr Danecek *et al.* SAMtools. https://github.com/samtools/samtools (2021).

27. UCSC Genome Browser. Genome Browser. https://genome.ucsc.edu/FAQ/FAQformat.html#format1.

28. Quinlan, A. R. & Hall, I. M. BEDTools: A flexible suite of utilities for comparing genomic features. *Bioinformatics* **26**, 841–842 (2010).

29. Bedtools. bedtools: a powerful toolset for genome arithmetic. https://bedtools.readthedocs.io/en/latest/index.html (2025).

30. SAMtools. *The Variant Call Format (VCF)*. https://samtools.github.io/hts-specs/VCFv4.2.pdf (2024).

31. Danecek, P. *et al.* The variant call format and VCFtools. *Bioinformatics* **27**, 2156–2158 (2011).

32. 1000 Genomes Project. *VCFtools*. https://vcftools.github.io/index.html (2025).

33. Ewing, B., Hillier, L., Wendl, M. C. & Green, P. Base-Calling of Automated Sequencer Traces Using Phred. I. Accuracy Assessment. *Genome Res* **8**, 175–185 (1998).

34. Ewing, B. & Green, P. Base-Calling of Automated Sequencer Traces Using Phred. II. Error Probabilities. *Genome Reasearch* **8**, 186–194 (1998).

35. Andrews, S. FastQC:  A Quality Control Tool for High Throughput Sequence Data. Available online at: https://qubeshub.org/resources/fastqc/about (2010).

36. Bolger, A. M., Lohse, M. & Usadel, B. Trimmomatic: A flexible trimmer for Illumina sequence data. *Bioinformatics* **30**, 2114–2120 (2014).

37. Martin, M. *Cutadapt Removes Adapter Sequences from High-Throughput Sequencing Reads. EMBnet Journal*. https://journal.embnet.org/index.php/embnetjournal/article/view/200/458 (2011) doi:https://doi.org/10.14806/ej.17.1.200.
